# Functional roles of purified yapsins from *Candida glabrata* (*Nakaseomyces glabratus*) in immune modulation and cross-species biofilm formation

**DOI:** 10.1038/s41598-025-15577-6

**Published:** 2025-09-01

**Authors:** Dorota Satala, Grzegorz Satala, Kamila Kulig, Justyna Karkowska-Kuleta, Andrzej Kozik, Maria Rapala-Kozik

**Affiliations:** 1 Department of Comparative Biochemistry and Bioanalytics, Faculty of Biochemistry, Biophysics and Biotechnology, Jagiellonian University, Gronostajowa 7, Kraków, 30-387 Poland; 2 Department of Medicinal Chemistry, Maj Institute of Pharmacology, Polish Academy of Sciences, Smetna 12, Kraków, Poland; 3 Department of Analytical Biochemistry, Faculty of Biochemistry, Biophysics and Biotechnology, Jagiellonian University, Gronostajowa 7, Kraków, 30-387 Poland

**Keywords:** *Candida glabrata*, Yapsin aspartic proteases, Host defense peptides, Immune modulation, *Galleria mellonella*, Biochemistry, Microbiology

## Abstract

*Candida glabrata* (currently classified as *Nakaseomyces glabratus*) is an opportunistic yeast-like fungus that causes infections in humans, with limited treatment options due to resistance to antifungal drugs. In contrast to *C. albicans*, which produces secreted aspartic proteases (Saps) involved in pathogenicity, *C. glabrata* expresses a distinct group of cell surface-associated aspartic proteases known as yapsins (Yps). While *YPS* gene deletion mutants have proposed roles in cellular homeostasis, their precise contribution to fungal virulence and host interactions remains unclear. Herein, we present the first detailed biochemical and functional characterization of two native Yps proteins, Yps3 and Yps9, purified from *C. glabrata* cultures. Both proteases displayed robust activity in a mildly acidic to neutral pH range (5.5–7.0), resistance to the classical aspartic protease inhibitor pepstatin A, and selectively degraded key host antimicrobial peptides, including LL-37 cathelicidin, histatin 5 (Hst5), and kininogen-derived peptide NAT26, by hydrolyzing lysine residues. Additionally, Yps9 promoted *C. albicans* biofilm dispersal. In a *Galleria mellonella* infection model, a pre-treatment with each protease enhanced larval survival and increased phenol oxidase activity, implying a role of yapsins in immune priming. Collectively, these findings reveal multifunctional roles for Yps3 and Yps9 in fungal virulence, biofilm modulation, and host immune interactions.

**Supplementary Information:**

The online version contains supplementary material available at 10.1038/s41598-025-15577-6.

## Introduction

Proteases, through their hydrolytic activity, play key roles in the pathogenesis of many microorganisms, including bacteria and fungi. These enzymes contribute to the breakdown of tissue barriers, inactivation of host defenses and acquisition of nutrients necessary for pathogen growth and survival^1–7^. Due to their multidirectional actions, proteases belong to the main factors that ensure high adaptive plasticity of microorganisms, enabling pathogen survival and spreading widely within the host^3–7^. In the case of pathogenic yeasts, one of the best-characterized proteolytic systems is a family of secreted aspartyl proteases (Saps) of *Candida albicans* – the most widespread pathogenic yeast^5,6^. *C. albicans* Saps comprise ten enzymes characterized by a wide range of substrate specificity, variable dependence of proteolytic activity on environmental conditions and variable levels of production in different types and stages of infection^5–7^. These proteases support host tissue invasion by degrading extracellular matrix proteins and components of the coagulation cascade, modulate the host immune response by impairing neutrophil and monocyte/macrophage function, play a role in biofilm structure formation, and mediate antifungal drug resistance^5,7^.

Of particular interest in the context of research on proteolytic virulence mechanisms is *Candida glabrata* (currently recommended name: *Nakaseomyces glabratus*)^8^ classified, depending on the region, as the second or third most common etiological agent of candidal infections^9^. Since the new nomenclature is not yet in widespread use, and the cited source data refer to *C. glabrata*, the former scientific name is used throughout this article for consistency. This species is distinguished from other *Candida* yeasts not only by its high resistance to azoles but also by a unique set of proteases belonging to the yapsin family (Yps). At least 11 different yapsin-encoding genes (*YPS1-11*) are present in the *C. glabrata* genome that, unlike the aforementioned *C. albicans* Saps, mostly have C-terminal sequences for putative GPI anchors, suggesting covalent attachment of the molecules to the cell surface^10^. To date, studies using deletion mutants have revealed yapsins’ involvement in the maintenance of cellular homeostasis, including the control of cell wall integrity under heat stress, survival in the presence of weak acids, including lactic acid, the maintenance of vacuole homeostasis and glucose homeostasis^11–13^. Importantly, emerging evidence indicate a direct involvement of yapsins in the colonization process and the long-term survival of the pathogen in the host. Namely, it was shown that a mutant lacking all *YPS* genes showed significant deficits in biofilm formation on abiotic surfaces, while re-expression of *YPS1* restored this ability^14^. In addition, the expression of *YPS* genes, particularly *YPS6* and *YPS7*, was dynamically modulated in response to environmental conditions that mimic host niches, such as saliva or vaginal environments^15^. In contrast, internalization of *C. glabrata* by immune cells resulted in an increased expression of *YPS2*, *YPS4*, *YPS5* and *YPS8*-*11* in macrophages, while an up-regulation of *YPS1*, *YPS2*, *YPS4-6* and *YPS8-11* was observed in neutrophils^16,17^. The mutant lacking all genes encoding *YPS* (*Cgyps1-11*Δ) was also shown to have a reduced survival and proliferation inside human and murine macrophages^14^. Moreover, it induced a stronger pro-inflammatory response in human THP-1 macrophages, as manifested by increased IL-1β production^14^. Additionally, through modulation of the p38 MAPK signaling pathway, it led to the downregulation of Arpc1B, resulting in the disorganization of actin filaments in host epithelial cells, reduced IL-8 secretion and impaired neutrophil migration^18^. Additionally, analyses in a murine model of systemic infection showed that the deletion of genes encoding *YPS* significantly impaired the ability of *C. glabrata* to colonize the kidney, liver, and spleen^14,16^leading to enhanced infiltration of immune cells within the kidney and increased production of pro-inflammatory cytokines, and limiting the spread of the pathogen in brain tissues^14^.

Despite the increasing recognition of the role of yapsin-like aspartyl proteases (*YPS*) in the survival and virulence of *C. glabrata*, much of the current knowledge derives from studies using gene deletion models. However, such models do not allow for direct conclusions regarding the biochemical activity, substrate specificity, or surface localization of the corresponding proteins, which are critical for understanding their mechanistic roles during infection.

Importantly, deletion mutants—while not suited for functional biochemical characterization—remain a valuable tool for dissecting immune responses in vivo. Their phenotypes often provide initial evidence of functional relevance, which can then be explored in more detail through biochemical studies. Nevertheless, the lack of direct evidence for whether the identified *YPS* proteases are produced and exposed on the fungal cell surface represents a major gap in our current understanding.

To address these limitations, the present study integrates both biochemical and functional approaches by analyzing two native Yps proteins, Yps3 and Yps9. We aimed to determine their enzymatic properties, their potential role in degrading host immune peptides, and their impact on fungal behavior such as biofilm dispersion. These data provide a necessary complement to genetic studies and contribute to a more comprehensive understanding of Yps proteases in *C. glabrata* pathogenesis.

## Results

### Isolation and biochemical characterization of Yps3 and Yps9

**Fig. 1:**
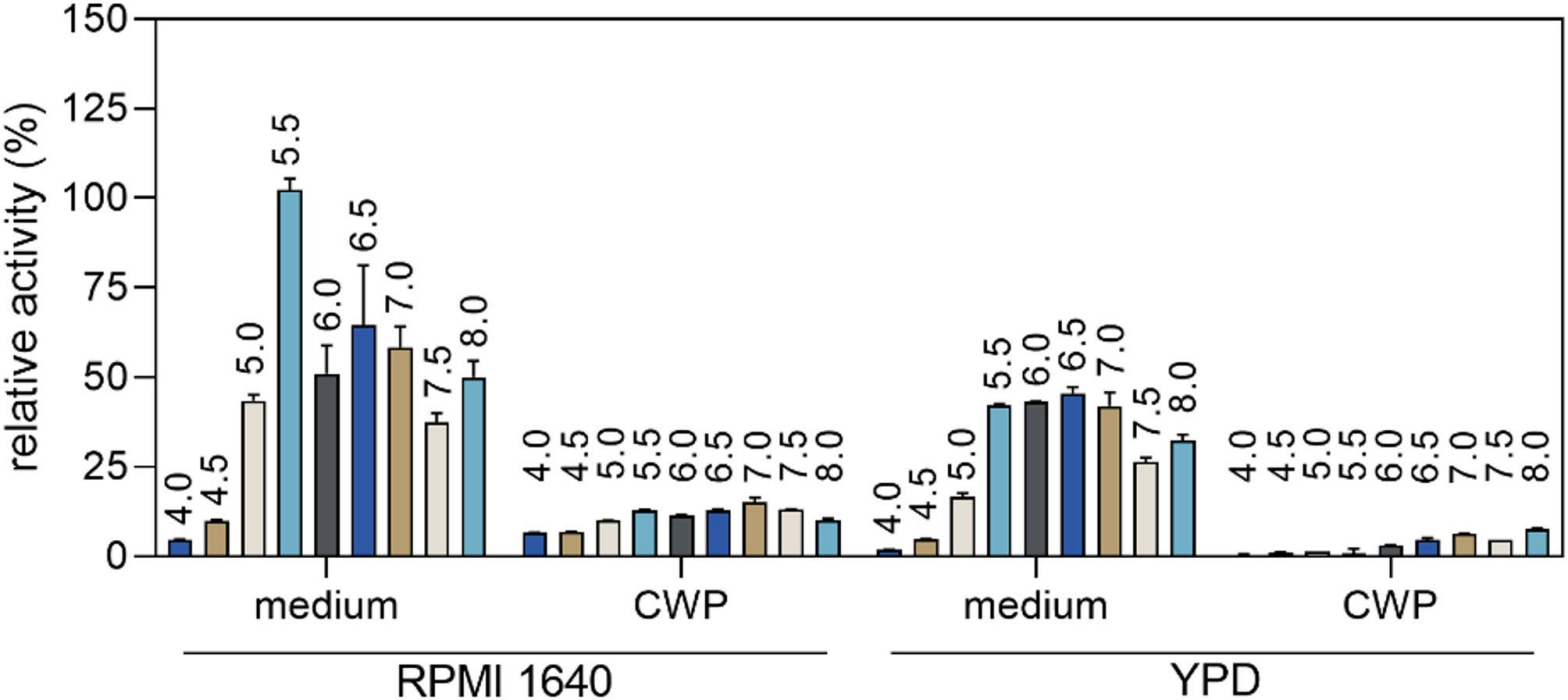
Dependence of the proteolytic activity of *C. glabrata* secretome and cell wall proteome on pH. Proteolytic activities of preparations containing a mixture of surface proteins (CWP) and concentrated post-culture media after culture in YPD broth or RPMI 1640 medium were determined using BODIPY-casein reagent. The results are presented as relative values, which were normalized against the highest recorded value, which was taken as 100%. The graph shows the averaged values obtained from the two experiments along with the standard deviation (SD).

According to the literature reports, Yps proteins can be found both on the cell wall and in the culture medium^19,20^. Therefore, in order to identify the best source for the purification of native proteins, we decided to carry out yeast culture under two standard conditions – in the YPD medium at 30 °C and in the RPMI 1640 medium at 37 °C. After growing the culture, we isolated cell wall proteins (CWP) from yeast cells using β-1,6-glucanase, while the post-culture medium was concentrated using a Spin-X UF concentrator. Since the activity of aspartyl proteases is often strongly influenced by environmental pH, we initially assessed the proteolytic potential of culture-derived protein fractions across a range of pH values to identify optimal conditions for enzyme activity. For the four preparations obtained – two CWP fractions and two post-culture media – we performed proteolytic activity determination using assay with casein derivative labeled with green-fluorescent BODIPY FL dye as the substrate (Fig. 1). Since we recorded the highest activity in RPMI 1640 post-culture medium, we chose this material as the source for further purification steps.

**Fig. 2:**
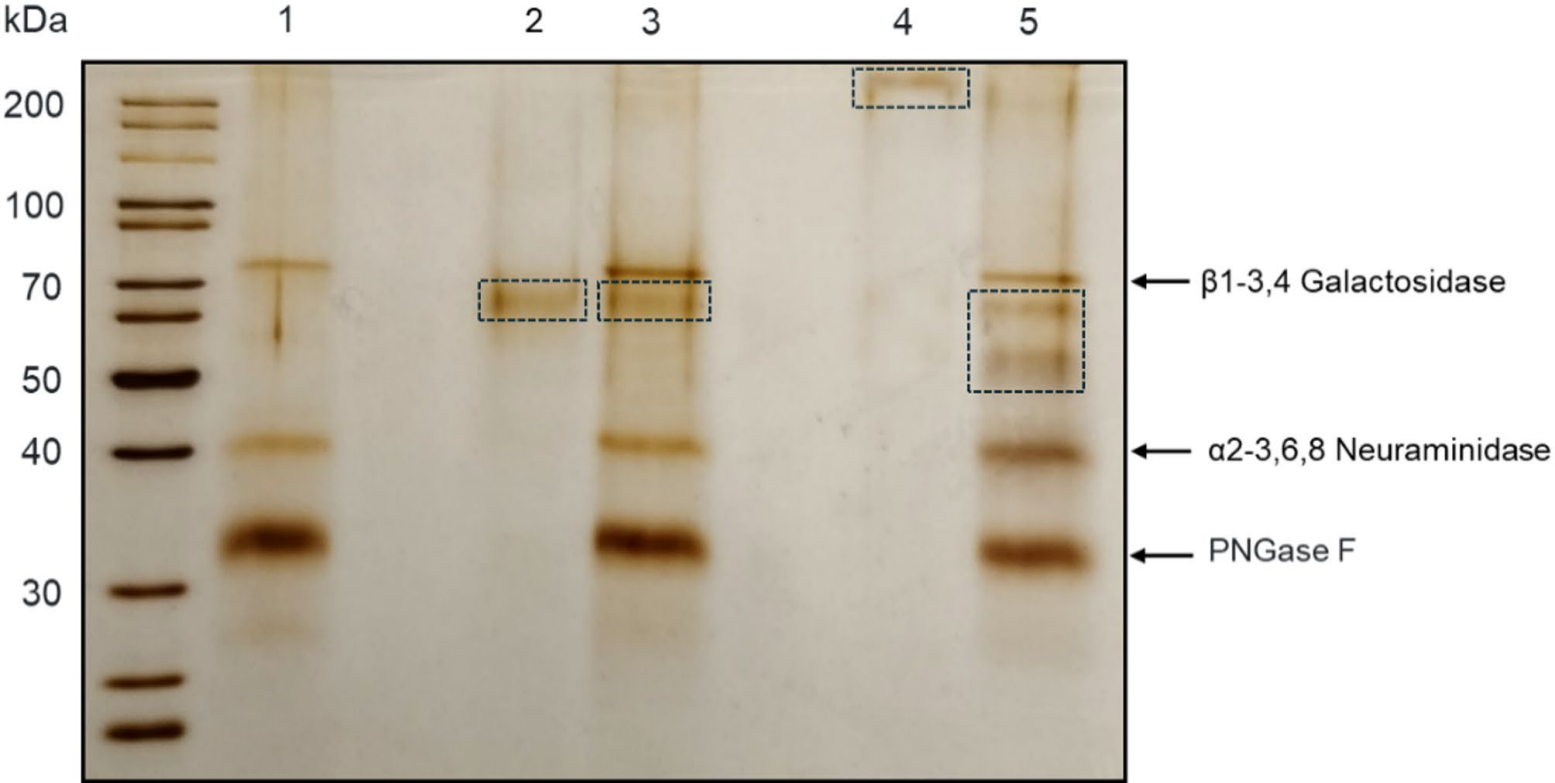
Electrophoretic characteristics of purified Yps of *C. glabrata*. Purified Yps3 (lane 2) and Yps9 (lane 4) proteins were separated by SDS-PAGE under reducing conditions using the Laemmli system and a 12% separating gel. The proteins were visualized by silver staining. Molecular weight marker mixture is shown in the left lane. In addition, electrophoretic patterns for Yps3 and Yps9 after treatment with the Protein Deglycosylation Mix are shown in lanes 3 and 5, respectively, with the control of the Protein Deglycosylation Mix itself shown in lane 1. Bands corresponding to both purified and deglycosylated proteins are indicated with dashed-line boxes.

The use of ion-exchange chromatography performed on a MonoQ column, followed by gel filtration on a Superdex200 column, allowed us to obtain protein preparations that presented single bands in the sodium dodecyl sulfate-polyacrylamide gel electrophoresis (SDS-PAGE) (Fig. 2; lanes 2 and 4), indicating high purity. Their identities as Yps3 and Yps9 was unambiguously confirmed using the liquid chromatography-coupled tandem mass spectrometry (LC-MS/MS) (Tab S1). Interestingly, the molecular weight of Yps9 protein observed on electrophoretic gel (about 200 kDa) significantly exceeded its theoretical molecular weight (59 kDa). The possibility that the high molecular weight of Yps9 may be due to extensive glycosylation of this protein, was verified using Protein Deglycosylation Mix (Fig. 2; see also Fig. S1 in the Supplementary Materials for a full-size electrophoresis image). A fading of the high-molecular-weight band in the electrophoretic gel along with an appearance of two new bands at significantly lower molecular weights (50 kDa and 60 kDa), confirmed the presence of glycans in Yps9 molecule but also suggested that under the applied experimental conditions deglycosylation was not complete. Additionally, LC-MS/MS analysis of these bands further supported the presence of Yps9 protein in both lower-molecular-weight forms.

A high susceptibility of Yps9 to N-glycosylation is explained by a supporting bioinformatic analysis using the NetNGlyc server^21^. As presented in Fig. S2 in the Supplementary materials, 15 potential N-glycosylation sites are identified in the Yps9 sequence, of which 6 show the highest prediction confidence. The most strongly predicted sites are located at positions 137 (NGTD), 166 (NLSN), 197 (NYSS), 290 (NITL), 324 (NGTV), and 360 (NVTD).

**Fig. 3:**
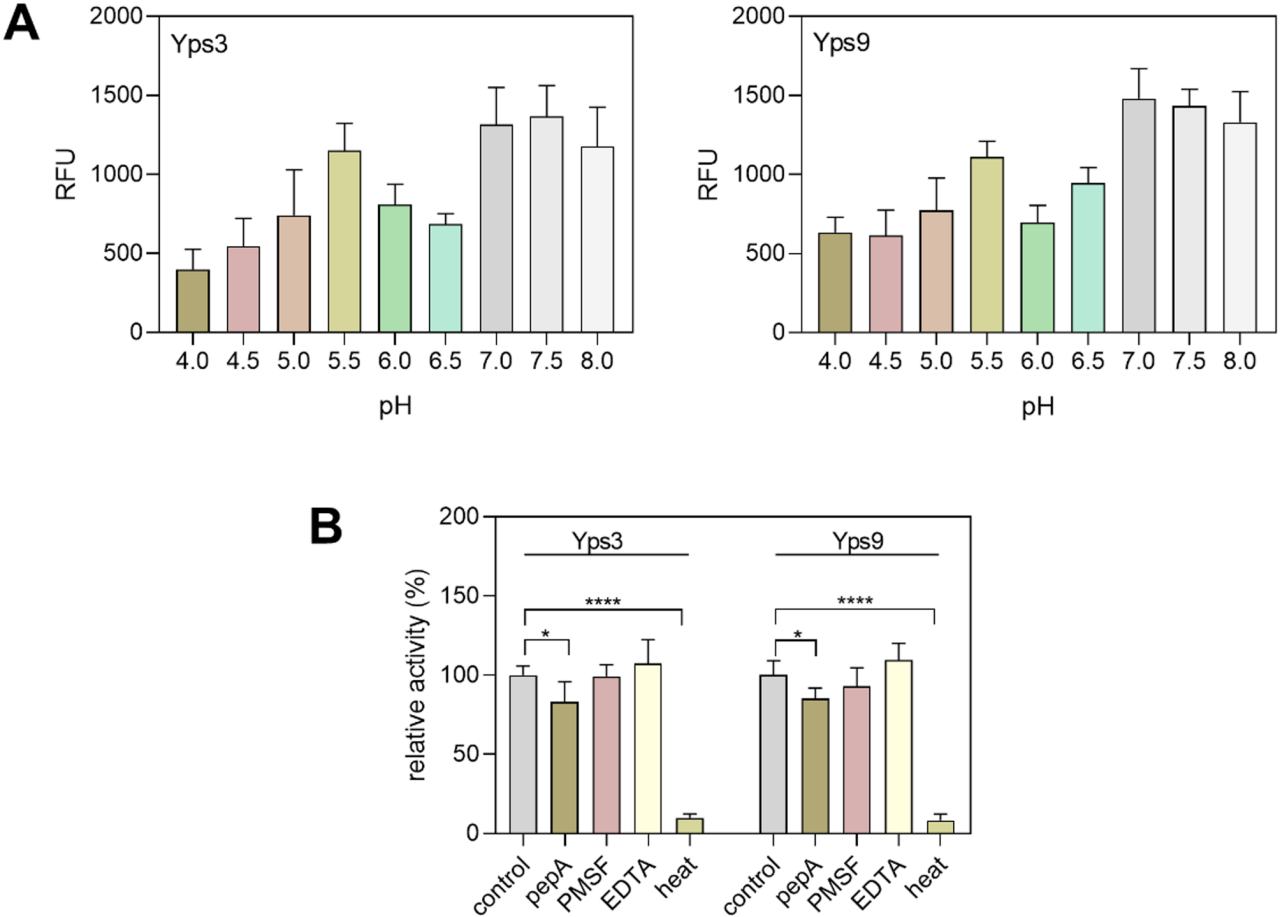
Dependence of proteolytic activity of purified Yps3 and Yps9 on pH (A). Proteolytic activity of Yps enzymes at pH 5.5 in the presence of inhibitors – pepA (final concentration 10 µM), PMSF (10 mM), and EDTA (5 mM) (B). Additionally, a sample was also prepared in which proteases were deactivated by heat treatment. The results obtained were normalized against the activity of Yps without inhibitors, which was taken as 100%. The graphs illustrate the averaged values obtained from two independent experiments along with the standard deviation.

In the next step of the study, we verified the proteolytic activity of the purified Yps proteins in a pH range of 4.0–8.0 using the BODIPY-casein assay (Fig. 3A). Since both Yps showed two peaks of proteolytic activity at pH 5.5 and 7.0, which was further maintained at slightly alkaline pH, we selected pH 5.5 and 7.0 for next analysis. Interestingly, pepstatin A (pepA), a classical aspartyl protease inhibitor, only modestly inhibited Yps at pH 5.5, with no significant inhibition of activity also observed when inhibitors of other protease classes such as phenylmethylsulfonyl fluoride (PMSF) and ethylenediaminetetraacetic (EDTA) were used (Fig. 3B). Interestingly, a SDS-PAGE electrophoretic analysis showed that bovine serum albumin (BSA), commonly used as a substrate for monitoring proteolytic activity, was not degraded at pH 5.5 and pH 7.0 even after overnight incubation (Fig. S3 in the Supplementary materials).

**Fig. 4:**
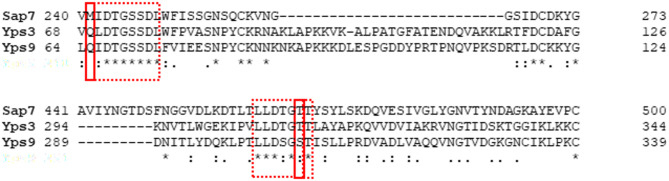
Comparison of amino acid sequence fragments of *C. glabrata* Yps3 (accession number Q6FVI0), *C. glabrata* Yps9 (accession number Q6FVH5), and C. *albicans* Sap7 (accession number P43096), aligned using Clustal Omega. The amino acid residues Met242 and Thr467 in the Sap7 sequence, suspected to restrict access to the binding site and contribute to the enzyme insensitivity to pepA inhibition, are highlighted, along with the corresponding residues in Yps3 and Yps9. For a full sequence alignment, see Fig. S3 in the Supplementary materials.

Among *C. albicans* Saps, Sap7 is insensitive to pepA inhibition, sharing this feature with yapsins. We thus hypothesized that some similarity between active centres of Sap7 and at least some yapsins, such as Yps3 and Yps9, may exist and that this similarity may be approached by bioinformatic analysis and molecular modelling. We first checked the overall similarities between Yps and Sap7 whole sequences (Tab. S2 in the Supplementary materials). As expected for proteins representing different families (yapsins vs. Saps), the sequence identity with Sap7 is as low as 33% and 32% for Yps3 and Yps9, respectively. Since it was suggested that Sap7 insensitivity to pepA may be due to the presence of Met242 and Thr467 residues that restrict access to the binding site^22^we analysed the amino acid sequence at the corresponding positions of Yps proteins (Fig. 4; Fig. S4 in the Supplementary materials). We showed that at positions corresponding to critical Sap7 residues, differences occur in Yps3 and Yps9, resulting in a Met to a Gln change at the first position and a Thr to Ser substitution in Yps9 at the second position (while Yps3 retains Thr). These positions are highlighted with a red box. Despite these substitutions, the surrounding region – outlined by a dashed line – displays a high degree of sequence similarity, suggesting functional or structural importance preserved among these proteins.

**Fig. 5:**
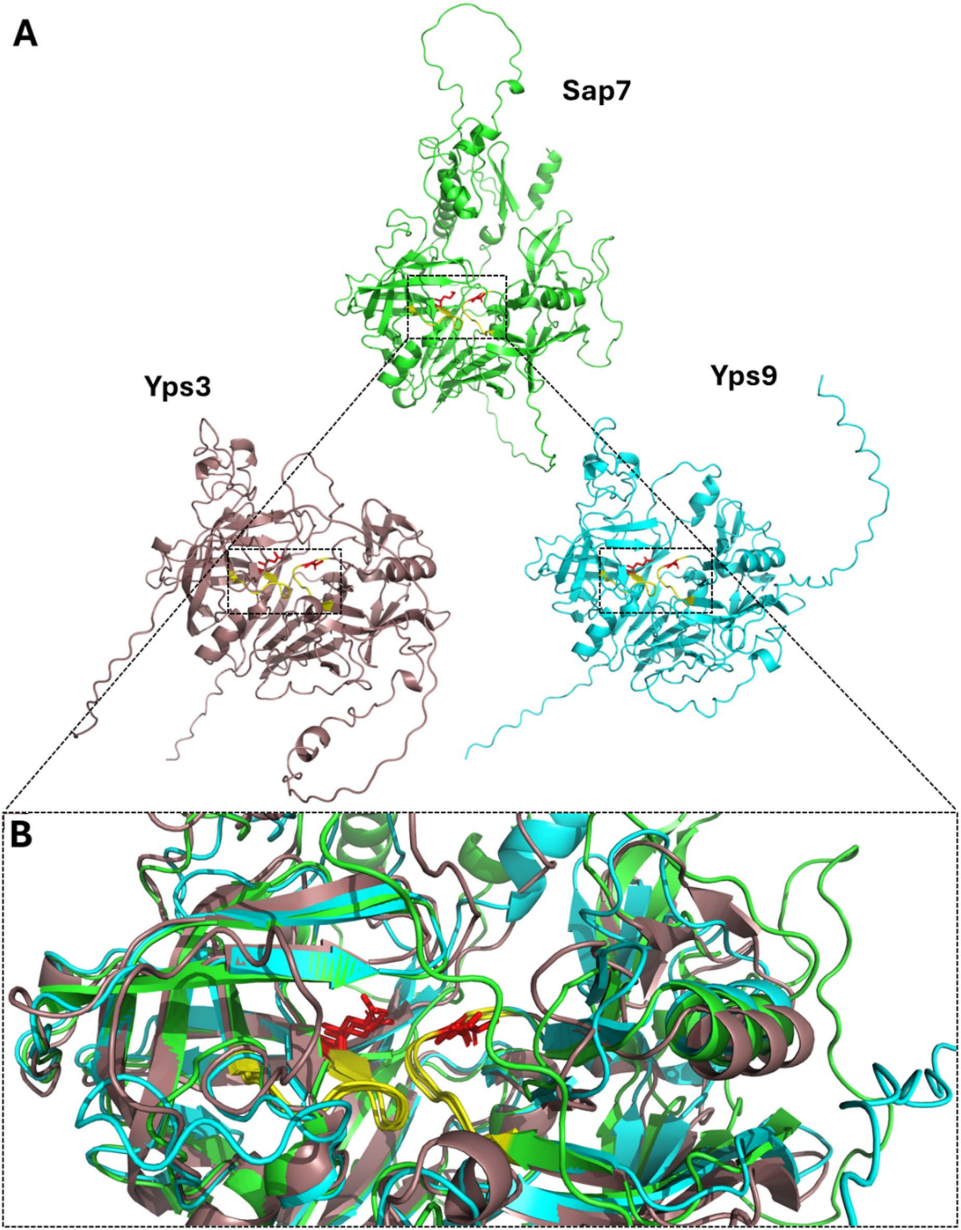
Structural comparison of Sap7, Yps3, and Yps9 proteases. (A) Individual AlphaFold structural models of Sap7 (AF-P43096-F1-model_v4, green), Yps3 (AF-Q6FVI0-F1-model_v4, light pink), and Yps9 (Q6FVH5-F1-model_v4, cyan). Residues previously identified as critical for pepA binding in Sap7 and their counterparts in Yps proteins are highlighted in red. The highly conserved region surrounding the active site (corresponding to the dashed-line region in Fig. 4) is highlighted in yellow. (B) Structural overlay of the predicted binding pocket region. Structural models were retrieved from the AlphaFold Protein Structure Database via UniProt (Varadi et al., 2022), optimized using the Protein Preparation Workflow (Maestro, Schrödinger LLC), and visualized with PyMOL v3.1.3 (Schrödinger LLC; https://pymol.org).

Following the above sequence analysis, we performed structural comparisons of Sap7, Yps3, and Yps9 using AlphaFold models (Fig. 5). Structural superposition revealed that, despite overall low sequence identity, the three-dimensional conformation of the region corresponding to the Sap7 pepA-binding site may be highly similar among these proteases. Importantly, even at positions displaying amino acid substitutions (Met vs. Gln for Sap7 vs. Yps3/Yps9; Thr vs. Ser for Sap7 vs. Yps9), spatial alignment demonstrates that the general topology and geometry of the active-site pocket remain nearly identical. These findings indicate that local structural features potentially critical for inhibitor binding are maintained, independent of broader sequence divergence observed across the proteins.

### Impact of Yapsins on biofilm structure

**Fig. 6:**
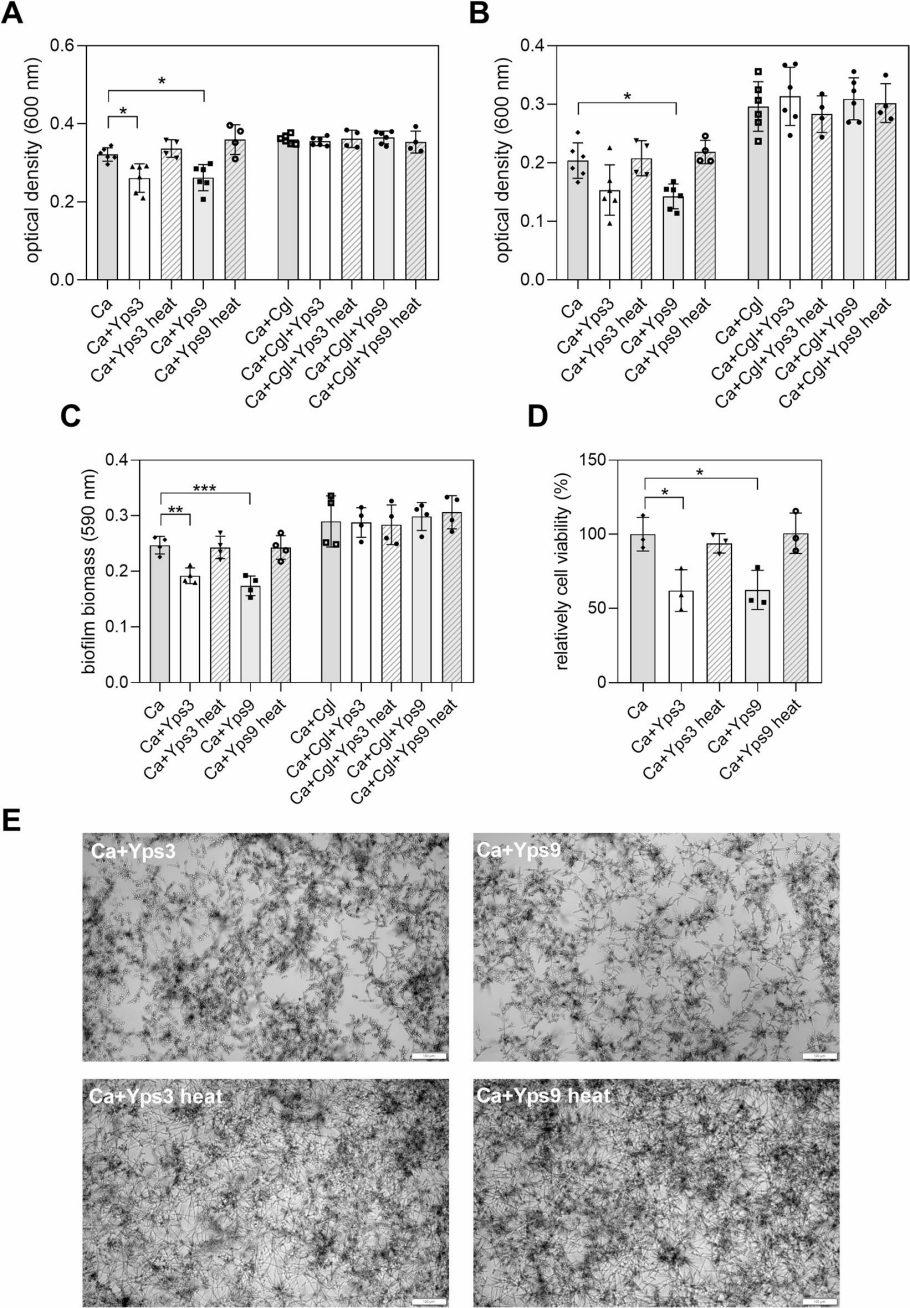
Evaluation of biofilm formation in the presence of purified Yps3 and Yps9 proteases and their heat-denatured controls. Following 24-hour incubation in the presence of native or heat-denatured Yps3 and Yps9 proteins, monospecies (*C. albicans*) and mixed (*C. albicans*–*C. glabrata*) biofilms were evaluated using complementary methods: OD_600_ was measured before (A) and after (B) PBS rinsing to estimate the amount of cells associated with the well surface. Crystal violet staining (C) was used to quantify total biofilm biomass, while the XTT reduction assay (D) was used to assess metabolic activity and is presented as a percentage of the untreated control. Transmitted light microscopy (E) was used to visualize representative wells. Biofilms cultured without Yps served as controls, while heat-denatured Yps3 and Yps9 were included as negative controls to confirm that the observed effects resulted from the proteolytic activity of native proteins. The statistical significance was determined by one-way ANOVA with Dunnett’s multiple comparison test using GraphPad Prism software and is marked with * *p* < 0.05, ** *p* < 0.01, *** *p* < 0.001.

In the clinic, biofilms as consortia in which many different species coexist have been shown to play an important role in the microbial virulence and mechanisms of drug resistance^23–25^. In the case of *C. glabrata*, among others, the possibility of synergistic interactions with *C. albicans* has been suggested, particularly in patients with advanced oral inflammation^26^. Considering reports suggesting that Yps proteases can affect Epa adhesins, which are responsible for *C. glabrata* surface adhesion and biofilm stability^16^ we investigated whether these proteases could also modulate *C. albicans* biofilm structure, either in a single-species or mixed systems. In the case of single-species *C. albicans* biofilm, treatment with Yps proteins reduced its growth; moreover, the biofilm detached more easily during rinsing with the effect depending on the type of protease used (Fig. 6). For Yps3, a minor dispersion was observed, although some tendency to weaken the biofilm was apparent, while for Yps9 the effect of biofilm reduction was statistically significant. Crystal violet staining and microscopic observation confirmed a reduction in biofilm biomass following treatment with native Yps proteins, whereas no such effect was observed in heat-denatured control samples. However, an XTT assay performed on monospecific *C. albicans* biofilms did not reveal significant changes in metabolic activity following treatment with Yps. Interestingly, no significant differences in protease-mediated degradation were observed in the mixed biofilm (Fig. 6, Fig. S5 in the Supplementary materials), indicating that the presence of *C. glabrata* may to some extent stabilize the *C. albicans* biofilm structure and reduce Yps proteolytic activity in biofilm dispersal.

### Modulation of the host immune response involving Yapsins

**Fig. 7:**
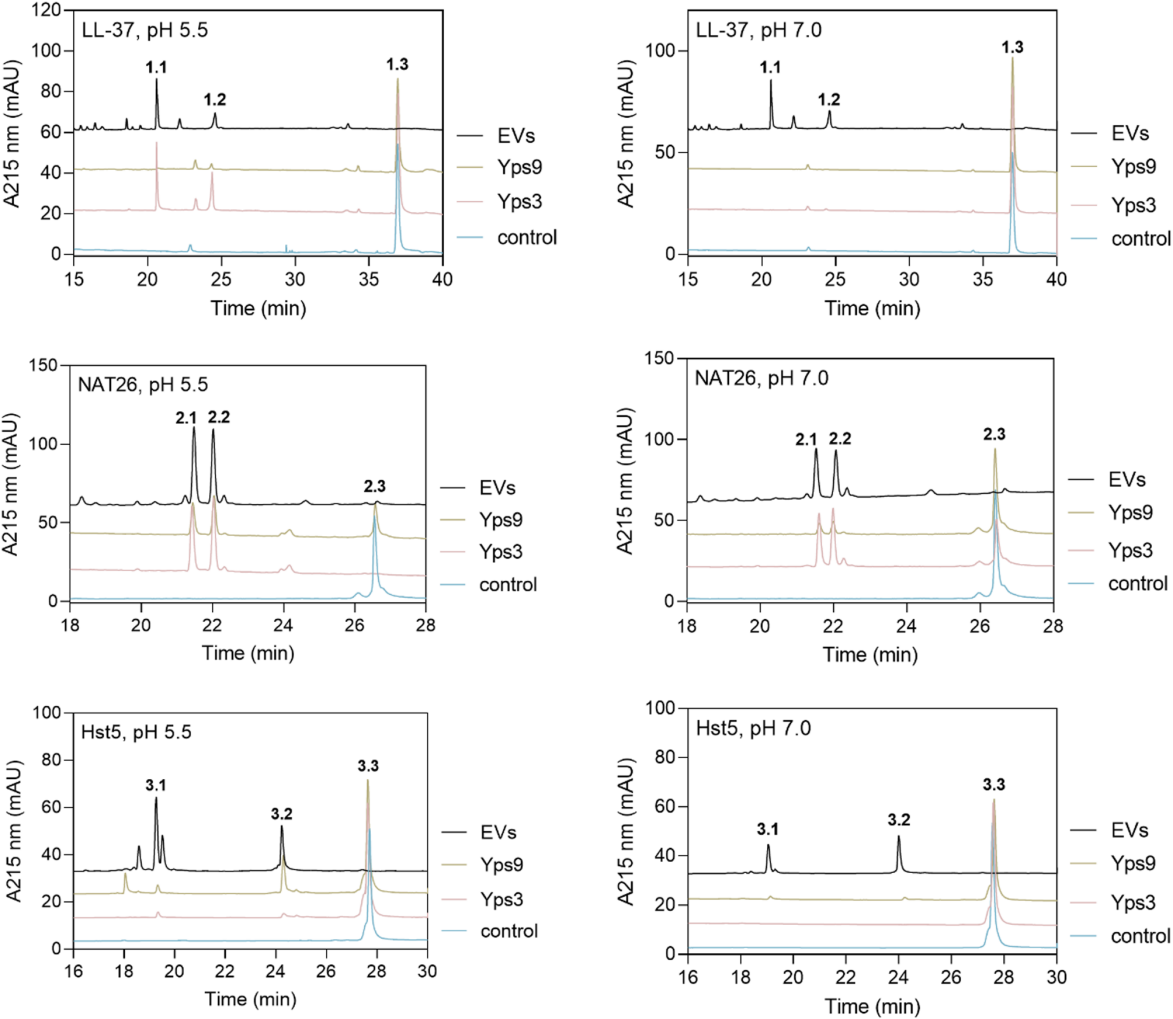
Chromatographic analysis of LL-37, Hst5 and NAT26 peptides after degradation by Yps3, Yps9 or EVs at pH 5.5 (left panel) and pH 7.0 (right panel). Numbered peaks correspond to the major degradation products that were analysed by tandem mass spectrometry (MS/MS).

One of the key biological effects reported for *C. albicans* Saps is their ability to degrade host proteins and peptides, resulting in the disruption of the host immune system allowing further development of the infection^6^. Therefore, in the next stage of this study, we tested an ability of isolated and purified Yps to degrade selected host peptides that demonstrate antifungal properties and are involved in host defense mechanisms during infection – cathelicidin LL-37, human kininogen-derived NAT26 peptide, and histatin Hst5^27,28^. We incubated each of the peptides with Yps3 or Yps9 at pH 5.5 and 7.0 for 5 h at 37 °C at a molar ratio of enzyme to substrate of 1:50, and then separated the resulting products by reversed-phase high-performance liquid chromatography (RP-HPLC). As shown in the representative chromatograms (Fig. 7), the proteolytic activity of Yps3 and Yps9 varies depending on pH and the type of substrate. Under acidic conditions (pH 5.5), Yps3 effectively degraded both LL-37 and NAT26, while showing no activity against Hst5. Under the same conditions, Yps9 exhibited other proteolytic preference, partially degrading NAT26 and fully degrading Hst5, but leaving LL-37 intact. At neutral pH (pH 7.0), Yps3 showed increased activity against NAT26, whereas LL-37 and Hst5 remained unaffected. Conversely, Yps9 exhibited only weak activity toward NAT26 at pH 7.0, with no degradation of the other peptides. To date, available proteomic studies have identified the presence of several yapsins also within *C. glabrata* extracellular vesicles (EVs), where they may be protected from degradation and less susceptible to inhibition. Thus far, Yps1, Yps3, Yps7 and Yps11 have been found in fungal EVs; however, further research is needed to identify additional yapsins within the vesicular proteome, thereby supporting the role of these structures as carriers encapsulating a diverse mixture of proteases^29–31^. Thus, we also decided to analyse the ability of these nanometer-sized, compositionally complex structures to hydrolyze human peptides. In our previous study we described the spherical shape and the size of *C. glabrata* EVs isolated from cells cultured under the same conditions as herein, and their average diameter of 171 nm^31^. In this study, *C. glabrata* EVs were freshly isolated from fungal liquid cultures and subsequently incubated with host antimicrobial peptides overnight at 37 °C, at pH 5,5 and 7, with 4 × 10^9^ EVs per sample. Then peptides were separated by HPLC. As shown in Fig. 7, EVs exhibited strong proteolytic activity against all tested host-derived peptides (LL-37, NAT26, and Hst5) at both pH 5.5 and 7.0, leading to their complete degradation. Importantly, the main degradation products were identical to those generated by purified Yps proteases, strongly suggesting that Yps presented within the EVs determine the observed overall proteolytic activity of vesicles. The main degradation products were identified by ESI-MS/MS, and their amino acid sequences are listed in Table 1.

**Table 1:** Amino acid sequences of host peptides generated from LL-37, Hst5 and NAT26 by Yps3 and Yps9, identified by tandem mass spectrometry (MS/MS).

No.	Calculated molecular mass (Da)	Sequence
LL-37		
1.1	1614.0	IGKEFKRIVQRIK
1.2	2182.9	LLGDFFRKSKEKIGKEFK
1.3	4491.8	LLGDFFRKSKEKIGKEFKRIVQRIKDFLRNLVPRTES
NAT26		
2.1	1477.8	ARVQVVAGKKYFI
2.2	1587.0	NATFYFKIDNVKK
2.3	3046.7	NATFYFKIDNVKKARVQVVAGKKYFI
Hst5		
3.1	892.2	HHSHRGY
3.2	1433.5	FHEKHHSHRGY
3.3	3036.3	DSHAKRHHGYKRKFHEKHHSHRGY

**Fig. 8:**
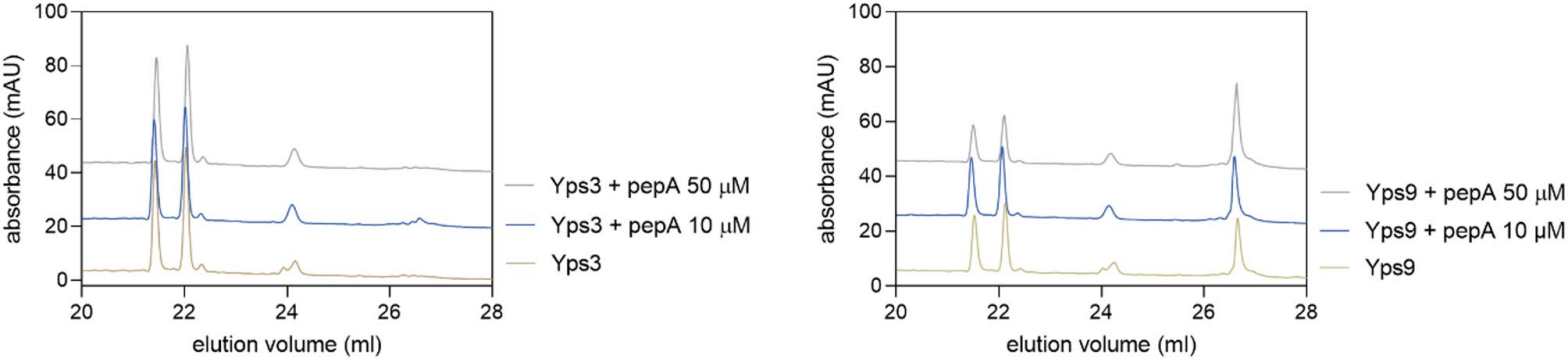
Chromatographic analysis of NAT26 peptide degradation by Yps3 (panel left) or Yps9 (panel right) at pH 5.5 without or with the presence of 10 µM or 50 µM pepA.

To confirm our previous results, indicating the insensitivity of Yps to pepA in the BODIPY-casein assay, we performed additional analyses of selected peptide degradation using the HPLC technique. For this purpose, we incubated the NAT26 peptide in a buffer of pH 5.5 in the presence of pepA in two concentrations – 10 µM and 50 µM. We did not observe a significant effect of the inhibitor on the activity of proteases, and only minor difference in the amount of substrate subjected to degradation and reduction in the amount of degradation products in the sample Yps9 + pepA 50 µM could be observed (Fig. 8).

**Fig. 9:**
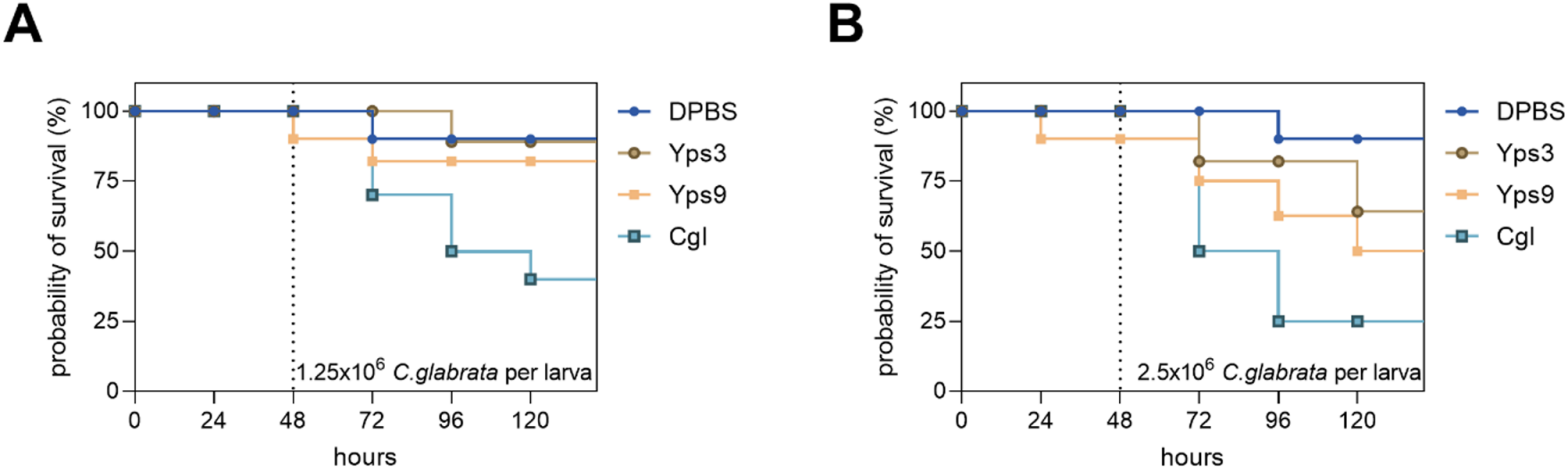
Effect of pre-injection with Yps proteins on the survival of *G. mellonella* larvae infected with *C. glabrata*. Larvae were injected with 1 µg of Yps3 or Yps9 in 10 µl, and 48 h later were challenged with two different doses of the pathogen ((A) 1.25 × 10⁶ or (B) 2.5 × 10⁶ CFU per larva). A dashed vertical line on the graph indicates the time point of pathogen injection (48 h post Yps-administration). Representative data are shown in the graph, with the experiment performed in three independent biological replicates, each group containing ten larvae. Survival rates were compared with the control group that received the pathogen without prior exposure to Yps. Larvae injected with DPBS served as an additional control.

To assess the effect of Yps3 and Yps9 proteases on the host immune response and their potential role in modulating *C. glabrata* infection, the survival rate of *G. mellonella* larvae after preincubation with these proteases and subsequent infection with the fungus was analysed (Fig. 9). In addition, the survival of larvae without exposure to the pathogen was monitored to determine the possible cytotoxicity of Yps3 and Yps9 (Fig. S5 in supplementary materials). As was found, injection of Yps3 and Yps9 did not cause significant larval mortality. Compared to the DPBS (Dulbecco’s Phosphate Buffered Saline) group, only a slight decrease in survival was observed, more pronounced in the case of Yps9, but after 120 h the differences were still insignificant and did not reach the level observed after *C. glabrata* infection. Analysis of larval survival after *C. glabrata* infection showed that in the DPBS control group, the survival rate remained high throughout the experiment, while larvae infected with *C. glabrata* showed significant mortality, especially in the later phase of infection. Pre-injection of larvae with Yps3 and Yps9 performed 48 h before initiation of larvae infection with *C. glabrata*, resulted in their higher survival rates compared to the group infected only with *C. glabrata*, suggesting that proteases may influence the course of infection or the host immune response. The protective effect was more pronounced at the lower infectious dose (1.25 × 10^6^ cells per larva), where larval survival in the presence of proteases remained similar to the DPBS control. At the higher dose (2.5 × 10^6^ cells per larva), the effect was attenuated, although still noticeable. There were no significant differences between the effects of Yps3 and Yps9, but the tendency toward protection was slightly more pronounced with Yps3. Taken together, the results indicate that Yps3 and Yps9 do not exhibit significant cytotoxicity against *G. mellonella* larvae, while the pre-injection with yapsins may reduce larval mortality after *C. glabrata* infection, especially at lower pathogen loadings.

**Fig. 10:**
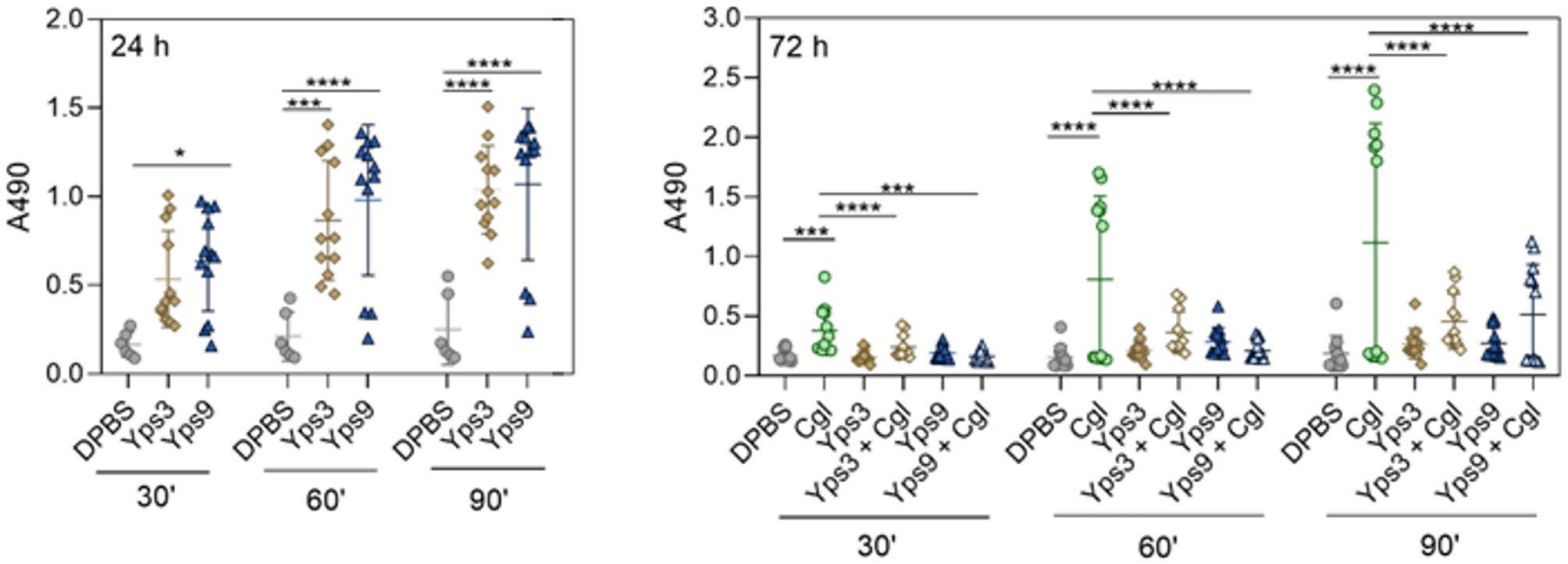
Kinetic measurement of phenol oxidase activity in the hemolymph of *G. mellonella* larvae pre-injected with Yps3 or Yps9 and subsequently infected with *C. glabrata*. Hemolymph was collected at three specific time points: 24 h after “immunopriming”, 48 h after “immunopriming” (immediately before infection), and 24 h after injection of live *C. glabrata* cells (corresponding to 72 h for “immunoprimed” larvae). The graph presents representative results from two independent experiments. In each group, hemolymph was collected from four larvae, and each measurement was performed in triplicate. Statistical significance was determined using ANOVA with Dunnett’s multiple comparison test using GraphPad Prism software and is marked with *p < 0.05, **p < 0.01, ***p < 0.001, ****p < 0.0001.

To assess the effect of Yps3 and Yps9 proteases on the host immune response, the activity of phenol oxidase, a critical invertebrate immune enzyme, was analysed 24 and 72 h after injection of Yps preparations (Fig. 10). Phenol oxidase activity was measured in the hemolymph of *G. mellonella* larvae after incubation on a microplate, with absorbance recorded at 30, 60, and 90 min to monitor reaction kinetics. At 24 h after pre-injection with yapsins, an increase in the enzyme activity was observed in groups of larvae treated with Yps3 and Yps9, suggesting a strong activation of the immune response, but the differences between Yps3 and Yps9 were not statistically significant. Subsequent measurements, taken 24 h after pathogen infection (72 h of the experiment), showed a significant reduction in enzyme activity in the groups pre-injected with Yps3 and Yps9 compared to the control group. This may suggest that the Yps proteins acted as immune activating factors, potentially inducing a form of “immune priming”, thereby protecting the larvae from further infection. Consequently, when *C. glabrata* infection occurs 48 h later, the immune response of the larvae is no longer manifested by elevated phenol oxidase activity, as immune mechanisms have been activated earlier and are likely to effectively control the infection.

## Discussion

In response to the growing threat of microbial resistance, in October 2022 the WHO classified *Candida glabrata* as a high-priority pathogen, underlining the need to dissect its virulence mechanisms and to intensify research on the interplay between pathogen virulence factors and infected host^32–36^.

Compared with the well-characterized Saps of *C. albicans*, the pathobiological contribution of Yps proteases in *C. glabrata* remains unclear owing to divergent activity reports. Current data on proteolytic activity in this species are inconsistent. Some studies detected no activity in blood or CVV isolates when BSA was used^37–39^ whereas others reported moderate activity in saliva^40^ and denture isolates^41,42^. These discrepancies suggest that variations in isolation environment and analytical methodology significantly impact the detection of proteolytic activity in *C. glabrata*. In the current work, we isolated native Yps and characterised their biochemical properties. Although the presence of Yps in the culture medium was rather inconsistent with their predicted anchoring to the cell wall, previous studies have confirmed that these enzymes can be secreted outside the cell. Analysis of the secretome of the vaginal isolate strain BG2 revealed the presence of Yps1, Yps3, Yps5-7, Yps9-11 in the medium after overnight culture in YNB broth (minimal yeast nitrogen base medium) at 30°C^19^. Moreover, secretome analysis of *C. glabrata* strain HLS119 cultured in synthetic complete (SC) medium without tryptophan at 37 °C showed the presence of Yps3, Yps6 and Yps9 as the main proteases secreted into the culture medium^20^. Finally, studies by Gonçalves et al.^43^ confirmed the presence of Yps2 and Yps5 in the biofilm matrix proteome of the ATCC 2001 strain. In our study, the secretome exhibiting the highest enzymatic activity contained two proteases – Yps3 and Yps9. The observed differences in the repertoire of Yps secreted outside the cell may be due to differences between strains or mechanisms regulating *YPS* expression.

In the study by Stead et al.^20^ using concanavalin A affinity chromatography and enzymatic digestion with endoglycosidase H, it was postulated that Yps3, Yps6 and Yps9 undergo the glycosylation – a posttranslational modification typical for proteins that passage via the classical secretory pathway. In our study, electrophoretic separation revealed that the molecular weight of purified Yps9 significantly exceeded theoretical predictions, which subsequent analyses attributed to glycosylation. In contrast, purified Yps3 exhibited a molecular weight consistent with theoretical expectations and lacked detectable glycosylation. As presented for *C. albicans*, the level of glycosylation of cell wall proteins can be affected by the availability of nutrients such as carbohydrates, nitrogen, or micronutrients^44^. Thus, it remains an open question whether usage of glucose-deficient RPMI 1640 medium affected the expression level and activity of glycosylation enzymes leading to a consequent decrease in the intensity of glycosylation of *C. glabrata* surface proteins, including Yps.

Analysis of the proteolytic activity of Yps3 and Yps9 showed that their optimum of activity is in the pH range of 5.5-8.0. Although for most aspartyl proteases the optimum of activity is in an acidic environment^45,46^. Interestingly, the optimum of Yps activity coincides with the pH found in the host niches most commonly inhabited by this species – the oral cavity^49,50^ and the vagina^51^. Moreover, in the analyses performed herein, Yps3 and Yps9 showed no susceptibility to inhibition by pepA, a classical aspartyl protease inhibitor. It has been suggested that Sap7 resistance to pepA may be due to the presence of Met242 and Thr467 residues, which restrict access to the inhibitor binding site^22^. Our structural analysis revealed substitutions at these critical positions in Yps3 and Yps9 – Met to Gln in both yapsins, and Thr to Ser in Yps9, but yet showing a remarkable spatial conservation of the active-site architecture across all three proteins. This suggests that the resistance to pepA likely arises not solely from these single-residue substitutions but may also involve more subtle local conformational features or dynamic properties of the inhibitor-binding pocket. A similar phenomenon has been reported for Aspergillopepsin II from *Aspergillus niger*, which possesses unconventional catalytic residues (Glu and Asp) and also remains insensitive to pepA^52,53^. In addition, we cannot exclude the possibility that extensive glycosylation of Yps9, and to a lesser extent Yps3, may contribute to partial masking of the active site and interfere with inhibitor binding. Although glycosylation is not typically expected to block enzymatic function, it may influence accessibility of small-molecule inhibitors. Further studies employing site-directed mutagenesis, deglycosylation under denaturing conditions, and broader inhibitor panels — including HIV protease inhibitors — may be needed to fully elucidate the inhibition profile of Yps proteases. Such analyses would also help clarify whether the observed resistance is an intrinsic structural feature or influenced by post-translational modifications. The biofilms play important roles in the mechanisms of fungal pathogen resistance against commonly applicable antifungal drugs, providing cells with significant protection through regulation of gene expression, structural modifications of the cell wall and changes in metabolic activity^54–56^. Importantly, in clinical settings, biofilms typically occur as multispecies structures, which are associated with increased morbidity and mortality^23–25,57^. Thus, *C. glabrata* is isolated simultaneously with *C. albicans* in about 80% of the population suffering from severe inflammation and poor oral hygiene^26^. In an in vitro study using reconstituted human vaginal epithelium (RHVE), a significant increase in colonization and invasiveness of *C. glabrata* was observed when it was growing together with *C. albicans*^58^. Moreover, a mixed biofilm of *C. albicans* and *C. glabrata* increased resistance to the antifungal drug caspofungin^59^ and reduced susceptibility to amphotericin B^25^; with the interaction between the species depending on a number of factors such as the strain type, cell number, or culture conditions^60^. In our analyses, we showed that the presence of purified proteases, especially Yps9, limits the growth of *C. albicans* biofilm and enhances its dispersion which may enable pathogens to disseminate to distant host sites and establish a new biofilm. Interestingly, it has recently been reported that EVs isolated from *C. glabrata* HTL strain, which according to proteomic studies produce Yps7, induce *C. albicans* Cht3 null mutant biofilm dispersion and fluconazole-induced biofilm reduction^30^. It should be noted that we did not observe a significant contribution of Yps to the disaggregation of mixed biofilm cells, highlighting the complexity of such a multispecies coexistence. One explanation is that the mixed biofilm may be stabilized by a physical mechanism, where significantly smaller cells of *C. glabrata* adhering to *C. albicans* filaments provide a cellular barrier to the action of Yps^59,61^. Another explanation could be a change in the composition of surface proteins in a mixed biofilm. As was shown, the coexistence of yeast can affect the increased gene expression of the adhesins *HWP1* and *ALS3* of *C. albicans*^59^ and the increased gene expression of the adhesins *EPA1*, *EPA8* and *EPA18* of *C. glabrata*^61^. Perhaps some adhesins are susceptible to the degradation by Yps, while others can bind them, leading to their inactivation.

*C. albicans* pathogenic yeasts have been shown to use proteolysis to deactivate key components of innate immunity, thus promoting the survival and spread of infection^6^. To assess whether *C. glabrata* Yps proteases play a similar role, we tested their hydrolytic activity against three host defense peptides relevant to innate immunity – LL-37, Hst5, and NAT26^62,63^. As shown, both proteases degrade all three peptides, with the highest efficiency observed at pH 5.5.

The distinct cleavage patterns suggest differences in the substrate specificity between Yps3 and Yps9—Yps3 preferentially targets LL-37 and NAT26, whereas Yps9 displays selective activity toward Hst5. Yps-mediated cleavage of NAT26 and Hst5 yielded fragments lacking antifungal activity, consistent with earlier findings for *C. albicans* Saps^27,28^. Thus, our findings indicate that Yps-mediated degradation of host peptides leads to the loss of antimicrobial properties, further supporting the role of these proteases in modulating host defence peptides. Interestingly, all identified cleavage products indicate a consistent preference of Yps proteases for hydrolysis after lysine residues, suggesting a conserved substrate recognition motif within this protease family.

Another hypothesis we explored was the possibility of host peptide degradation by EVs. Recently published data indicated that EVs isolated from *C. albicans* are capable of degrading NAT26 and the resulting fragments are analogous to those observed for the action of individual Saps^65^. Our analysis showed that *C. glabrata* EVs degrade all peptides tested, with Yps proteases appearing to play the major role in the degradation of LL-37, NAT26, and Hst5, as the main cleavage products generated by EVs were identical to those produced by purified Yps proteases. This finding suggests that EVs may efficiently deliver proteolytic enzymes, enabling targeted inactivation of host defense peptides in the extracellular space. This mechanism may enhance immune evasion and promote persistence.

In the *G. mellonella* model, we found that the administration of purified Yps is not toxic to the host, and that prior exposure to these proteases makes the larvae more resistant to subsequent infection. One of the key mechanisms of this response appears to be the activation of the melanization pathway, in which phenol oxidase contributes to host defense^66–68^. In our study, we noted a significant increase in phenol oxidase activity in hemolymph 24 h after administration of Yps. However, after next 48 h, pathogen administration was no longer associated with a further increase in enzyme activity. Importantly, larvae that had not been previously exposed to Yps showed clear signs of inflammation. This may suggest that prior exposure to Yps induces an adaptive immune response mechanism, leading to earlier activation of the melanization pathway, which increases the immune readiness of the larvae. Consequently, pre-stimulation of the enzyme may allow more efficient elimination of the pathogen without the need for strong activation of phenol oxidase. In contrast, the lack of prior contact with Yps causes the larval immune system to respond with a delay, leading to the development of inflammation in response to infection.

The results presented herein provide new data on the role of *C. glabrata* Yps proteases in virulence and host interaction mechanisms. We demonstrated that these enzymes can affect biofilm structure, promoting biofilm dispersion by which may have important implications for the pathogenesis of *C. glabrata* infections, especially regarding mixed biofilm formation. In addition, analysis of the proteolytic activity of Yps revealed their ability to degrade peptides involved in innate immunity, which may be one of the mechanisms of immune response evasion. Importantly, in vivo studies using a *G. mellonella* model indicate that prior exposure to Yps leads to an increase in larval resistance to subsequent infection, suggesting a potential effect of these proteases on modulating the immune response. These findings underscore the importance of further research of the function of yapsins in *C. glabrata* infections and their potential use in immunization and infection prevention strategies, particularly in the light of their dual role in modulating host immunity and influencing fungal virulence. As such, Yps proteases may hold considerable promise as novel targets for the development of future antifungal therapies.

## Methods

### Yeast strain and materials

The *C. glabrata* strain CBS138 (ATCC^®^ 2001™), originally isolated from human feces, was obtained from the American Type Culture Collection (Manassas, VA) and used in this study. Commercial antimicrobial peptides – LL-37 (LLGDFFRKSKEKIGKEFKRIVQRIKDFLRNLVPRTES), His5 (DSHAKRHHGYKRKFHEKHHSHRGY), and NAT26 (NATFYFKIDNVKKARVQVVAGKKYFI) – were purchased from Sigma-Aldrich (St. Louis, MO, USA), Eurogentec (Liege, Belgium), and Lipopharm (Zblewo, Poland), respectively.

### Culture conditions

*C. glabrata* was cultured in YPD medium (1% yeast extract, 2% soybean peptone, and 2% glucose) at 30 °C for 18 h or in defined RPMI 1640 medium (pH 7.4) at 37 °C for 24 h. The cultures were incubated with shaking at 170 rpm (MaxQ 4000, Thermo Fisher Scientific, Waltham, MA, USA). After incubation, yeast cells were separated from the culture medium by centrifugation (3,000 × g, 3 min). The cells were then treated with β-1,6-glucanase following a previously described method^69^ to obtain a mixture of cell wall proteins (CWP). Subsequently, after a two subsequent centrifugations (6,000 × g, 6 min; 10,000 × g, 10 min), the medium was concentrated 20-fold using a Spin-X UF concentrator (Corning, New York NY, USA). The obtained CWP proteome and secretome preparations were dialyzed against PBS for 48 h or against 20 mM Tris, pH 8.0 if they were subjected to protein purification.

### Proteolytic activity and Inhibition of Yps3 and Yps9

Proteolytic activity was assessed using the EnzChek™ Protease Assay Kit (Thermo Fisher Scientific), in which protease-catalysed hydrolysis releases a highly fluorescent dye, leading to an increase in fluorescence intensity proportional to protease activity. According to the manufacturer’s instructions, the sample (10 µl of CWP or concentrated culture medium normalized for protein concentration, or a 10 nM solution of Yps3 or Yps9) was suspended in 100 µl of the appropriate buffer (50 mM acetate buffer for pH 4–5.0 or 50 mM phosphate buffer for pH 6–8) with 1 µg of BODIPY FL-labelled casein. The reaction mixtures were incubated in a black, flat-bottom, 96-well microtiter plate (Greiner Bio-One, Kremsmünster, Austria) at 37 °C in the dark for 24 h. Fluorescence intensity was then measured using a Synergy H1 microplate reader (BioTek, Winooski, VT, USA) at excitation and emission wavelengths of 485 and 528 nm, respectively.

Additionally, experiments were conducted for Yps3 and Yps9 in the presence of different inhibitors. For this purpose, pepA, PMSF and EDTA were added to the reaction mixture containing purified proteases suspended in acetate buffer (pH 5.5) at final concentrations of 10 µM, 10 mM and 5 mM, respectively. Casein FL-labelled was then introduced, and the assay was carried out as described above. Control samples, prepared without inhibitors, were heat-inactivated by boiling for 30 min in an Eppendorf tube before being transferred to the microplate wells.

### Purification of Yps3 and Yps9

Concentrated, predialyzed RPMI 1640 medium was applied to a Mono Q HR 5/5 1 ml column (Pharmacia Biotech, NJ, USA) pre-equilibrated in 20 mM Tris, pH 8.0, at a flow rate of 1 ml/min. Separations were performed using an ÄKTA Pure chromatograph system (GE Healthcare, Uppsala, Sweden). Bound proteins were eluted from the column using a 60 ml linear gradient of 0–1 M NaCl in 20 mM Tris buffer, pH 8.0. The collected fractions (1 ml) were analysed by SDS-PAGE and protease activity measurement. The selected fractions were loaded onto a Superdex 200 h 10/30 column (Amersham Bioscience, Uppsala, Sweden), equilibrated in 50 mM sodium phosphate buffer, pH 7.4, containing 150 mM NaCl. Protein separation was performed at a flow rate of 0.5 ml/min. Yps purity and identity were confirmed by SDS-PAGE and LC-MS/MS analysis according to a previously published protocol^70^.

### Glycosylation analysis

The presence of glycans on purified Yps was analysed using a Protein Deglycosylation Mix (New England Biolabs, Ipswich, MA, USA), according to the manufacturer’s instructions. The enzymes included in the kit are capable of removing all N-glycans and simple O-glycans, as well as some complex O-glycans.

### Alignment of the amino-acid sequences of *C. glabrata* Yps3 and Yps9 and *C. albicans* Sap7

Fungal protease sequences were aligned and analysed using Clustal Omega (1.2.4)^71^. The accession numbers of *C. glabrata* Yps sequences are A0A3G1PW07, Q6FVJ4, Q6FVI0, Q6FVH9, Q6FVH8, Q6FVH7, Q6FY32, Q6FVH6, Q6FVH5, Q6FVH4, Q6FVH3, while the accession numbers of *C. albicans* Sap sequences are P0CY26, P0CS83, P0CY28, Q5A8N2, P43094, P43095, P43096, O42778, O42779, Q5A651.

### Molecular modelling

Structural models of selected proteases were retrieved from the AlphaFold Protein Structure Database via UniProt^72^. The following models were used in the study: Sap7 (AF-P43096-F1-model_v4), Yps9 (AF-Q6FVH5-F1-model_v4), and Yps3 (AF-Q6FVI0-F1-model_v4). Prior to visualization and analysis, all structures were optimized using the Protein Preparation Workflow available in the Maestro software package (Schrödinger, LLC, New York NY, US). Structural figures were generated using PyMOL v3.1.3 (Schrödinger LLC; https://pymol.org).

### Biofilm formation in the presence of Yps3 and Yps9

In the initial phase of biofilm formation, *Candida* cells (2 × 10⁵ or 1 × 10⁵ *C. albicans* and 1 × 10⁵ *C. glabrata*) were suspended in 100 µl of RPMI 1640 medium containing phenol red and buffered with 25 mM HEPES (Biowest, Bouchemaine, France). The suspension was incubated for 90 min at 37 °C under 5% CO₂ and 95% humidity in a Corning^®^ 96-well black/clear flat-bottom polystyrene high-bind microplate (Corning). Following incubation, the culture medium, along with non-adherent cells, was carefully removed, and the wells were rinsed once with 100 µl of PBS. Subsequently, purified Yps proteins were introduced into the wells at a final concentration of 100 nM in 200 µl of fresh RPMI 1640 medium buffered with 25 mM HEPES. The biofilm was then allowed to develop over the next 24 h under the same incubation conditions. For the control biofilm, fungal cells were cultured following the same protocol but without exposure to Yps proteins or in the presence of heat-denatured Yps3 and Yps9 (boiled for 30 min), to distinguish effects related to proteolytic activity from non-specific protein exposure. To assess biofilm formation and structure, several complementary methods were employed, including optical density of biofilms (OD) measurement, microscopy, crystal violet staining, and the XTT reduction assay. After 24-hour incubation with or without native or heat-denatured Yps proteins, the OD was measured at 600 nm using a nine-point area scan method (Synergy H1 microplate reader, BioTek). The wells were then washed three times with 200 µl of PBS, and OD was measured again. Background values from control wells without biofilm were subtracted. Immediately after the OD measurement, representative wells were imaged using transmitted light microscopy (Olympus IX73 inverted microscope) to visualize general biofilm morphology. Biofilm biomass was then quantified using crystal violet (CV) staining. For this purpose, the wells were incubated with 0.5% CV (Sigma-Aldrich) for 15 min at room temperature, washed three times with distilled water (300 µl), and destained with 80 µl of 70% ethanol for 15 min. From each well, 50 µl of the extracted dye was transferred to a new microplate, and absorbance was measured at 590 nm. Metabolic activity was assessed using the XTT (sodium 3′-[1-(phenylaminocarbonyl)-3,4-tetrazolium]-bis (4-methoxy6-nitro) benzene sulfonic acid hydrate) assay (Thermo Fisher Scientific) after 40 min of incubation at 37 °C, followed by transfer of 100 µl supernatant to a fresh plate and absorbance measurement at 450 nm. In all assays, heat-denatured Yps proteins served as negative controls to confirm that the observed effects were related to proteolytic activity.

### Isolation of *C. glabrata *EVs

The cultures of *C. glabrata* were routinely performed in YPD medium for 18 h at 30 °C with an orbital rotary shaking at 170 rpm. Then 4 × 10^10^ cells were inoculated into 400 ml of RPMI 1640 medium for 72 h at 37 °C (170 rpm). Then EVs were harvested from the supernatants from 72-h culture after discarding fungal cells as previously described^31^. Briefly, the centrifugation at 4,000 × g for 15 min at 4 °C was repeated twice to discard cells and cellular debris. The obtained supernatants were concentrated 400-fold using an Amicon Ultra-15 Centrifugal Filter Unit with a 100 kDa cut off (Merck, Darmstadt, Germany). Next, samples were centrifugated at 5,000 × g for 5 min at 4 °C. The supernatant obtained after discarding the pellet was filtered using an Ultrafree-CL Centrifugal Filter with pore size of 0.65 μm (Merck). The ultracentrifugation at 40,000 rpm (which corresponds to relative centrifugal field of 146,000 × g with *k* factor 108) for 1 h at 4 °C of samples containing EVs was performed, using a fixed-angle type 50.2 Ti Rotor and polycarbonate thick wall centrifuge tubes (13 × 64 mm) with 13 mm diameter Delrin tube adapters in an Optima™ L-90 K Ultracentrifuge (Beckman Coulter, Indianapolis, IN, USA). The pellet with EVs was transferred in 200 µl of 0.22 μm-filtered PBS buffer (pH 7.4) to Eppendorf tubes and divided into 20 µl portions which were frozen at -80 °C for further use.

### Analysis of antimicrobial peptide degradation by RP-HPLC and mass spectrometry

Antimicrobial peptide degradation under the influence of purified Yps proteins was analysed following previously published protocols^27,64^. Specifically, a 100 µl reaction mixture was prepared in an Eppendorf tube, containing 5 µM host-derived peptide and 20 nM Yps, maintaining an enzyme-to-substrate ratio of 1:50 in either acetate buffer (pH 5.5) or phosphate buffer (pH 7.0). The mixtures were incubated at 37 °C for 5 h, after which 20 µl of 1 M NaCl was added. Samples were briefly incubated on ice and centrifuged at 13 000 × g for 10 min. The resulting supernatants, containing peptide fragments, were analysed using RP-HPLC on a Dionex Ultimate 3000 system (Thermo Scientific). Peptide separation was performed on a Eurosil Bioselect 300-5 C18 column (5 μm, 4 mm × 250 mm) (Knauer, Berlin, Germany) using a binary solvent system: solvent A (0.1% TFA in water) and solvent B (0.08% TFA in 80% acetonitrile) (Merck). The flow rate was set at 1 mL/min, with spectrophotometric detection at 215 nm. Peptide fragments were separated under the following gradient conditions: 0–25% solvent B over 31 min for Hst5, 10–70% over 41 min for LL-37, and 10–50% over 36 min for NAT26. The major peaks obtained in the chromatograms were collected for further analysis. Additionally, for NAT26 at pH 5.5, a control reaction was conducted in which the reaction mixture contained both the enzyme and substrate, with the addition of 10 µM pepA as a potential enzyme inhibitor to evaluate its effect on peptide degradation. Moreover, the ability of EVs to degrade host peptides was also evaluated. For this purpose, 4 × 10^9^ EVs per sample were added to the reaction mixture and incubated for 24 h under the same conditions. After mixing with 1 M HCl and centrifugation at 13 000 × *g* for 15 min supernatants were passed through 0.22 μm filters (Sigma-Aldrich).

Fractions from RP-HPLC were evaporated and reconstituted in 30% methanol with 0.1% formic acid, an then analysed using an HCTultra ETDII mass spectrometer (Bruker, Bremen, Germany). Samples were introduced via direct infusion at 180 µl/h into an electrospray ionization (ESI) source operating in positive ion mode. Peptide identification was performed using DataAnalysis™ 4.0, Biotools™ 3.2 (Bruker), and an in-house Mascot server (Matrix Science, UK), searching against the Swiss-Prot database.

### In vivo assessment of Yps3 and Yps9 effects on *G. mellonella* survival and phenol oxidase activity

The in vivo effects of Yps3 and Yps9, as well as their potential impact on *C. glabrata* infections, were evaluated using commercially purchased *G. mellonella* larvae. Four experimental groups were established, each consisting of ten randomly selected larvae. In the first group, larvae were inoculated in the last left proleg with 10 µl of a solution containing 1 µg of purified Yps3 and Yps9 using a 10 µl Hamilton syringe (Merck). In the second group, larvae received an initial injection of Yps3 and Yps9 into the left proleg, followed by a second injection of *C. glabrata* after 24 h. The fungal inoculum was administered in two different concentrations: 1.25 × 10^6^ or 2.5 × 10^6^ cells per larva. The third group received only *C. glabrata* at the same two concentrations, while the fourth control group was injected with 10 µl of sterile DPBS. Following inoculation, *G. mellonella* larvae were maintained at 37 °C in Petri dishes and monitored for 120 h. Mortality was assessed every 24 h based on dark pigmentation and the absence of movement upon physical stimulation. Three independent biological replicates were conducted to ensure reproducibility of the results.

Hemolymph for the phenol oxidase activity assay was collected by puncturing the head with a sterile needle 24 h after the injection of DPBS or *C. glabrata* at a dose of 2.5 × 10^6^ cells per larva. Additionally, hemolymph was collected from larvae that had been pre-injected with purified Yps3 and Yps9 24 h earlier and 24 h after pathogen administration. The assay was performed as described by Sulek et al.^67,68^. Briefly, 2 µl of freshly collected hemolymph was mixed with 20 µl of Tris-HCl buffer (50 mM, pH 7.4) containing 150 mM NaCl and 5 mM CaCl₂, followed by a 20-minute incubation at room temperature in a 96-well microplate. Next, 180 µl of 2 mM L-DOPA in phosphate buffer (pH 6.5) was added, and absorbance at 490 nm was recorded every 5 min for 180 min at 37 °C using a Synergy H1 microplate reader with linear shaking. The experiment was conducted in at least three biological replicates, with four larvae per group. A water-only control was included, and its absorbance values were subtracted from the sample readings.

### Statistical analysis

Statistical analysis was performed using GraphPad Prism 8 (GraphPad Software, San Diego, CA, USA). Statistical significance was assessed by an unpaired t-test and one-way ANOVA followed by a Dunnett’s test for multiple comparisons.

## Supplementary Information

Below is the link to the electronic supplementary material.

Supplementary Material 1

## Data Availability

The datasets used and/or analysed during the current study available from the corresponding author on reasonable request.
