# Supplementary material for: Functional roles of purified yapsins from Candida glabrata (Nakaseomyces glabratus) in immune modulation and cross-species biofilm formation
Source: Sci Rep. 2025 Sep 1;15:32115. doi: 10.1038/s41598-025-15577-6 (PMC12402140; doi:10.1038/s41598-025-15577-6)
Supplement: Supplementary file 1 — Supplementary Material 1 [file 41598_2025_15577_MOESM1_ESM.pdf]

SUPPLEMENTARY MATERIALS

Functional roles of purified yapsins from *Candida glabrata* (*Nakaseomyces glabratus*) in immune modulation and cross-species biofilm formation

Dorota Satala[1]*, Grzegorz Satala[2], Kamila Kulig[1], Justyna Karkowska-Kuleta[1], Andrzej Kozik[3], Maria Rapala-Kozik[1]

[1]Department of Comparative Biochemistry and Bioanalytics, Faculty of Biochemistry, Biophysics and Biotechnology, Jagiellonian University, Gronostajowa 7, 30-387 Kraków, Poland

[2]Department of Medicinal Chemistry, Maj Institute of Pharmacology, Polish Academy of Sciences, Smetna 12, Kraków, Poland

[3]Department of Analytical Biochemistry, Faculty of Biochemistry, Biophysics and Biotechnology, Jagiellonian University, Gronostajowa 7, 30-387 Kraków, Poland

* Correspondence: phone number: 48126646524; e-mail: dorota.satala@uj.edu.pl

A

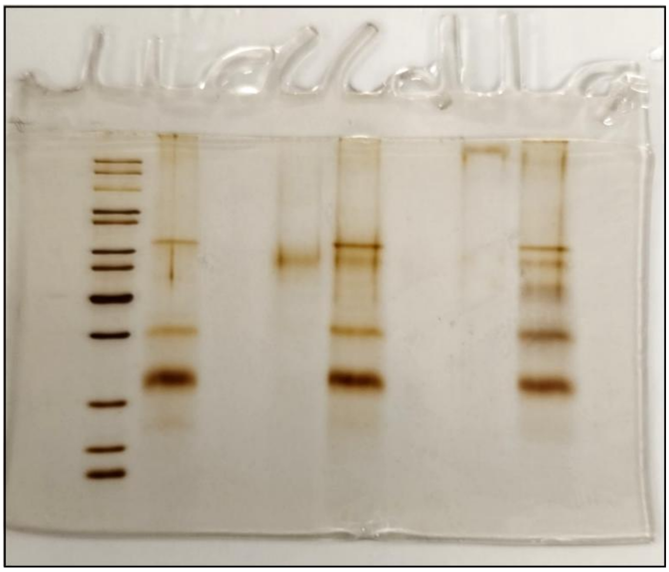

B

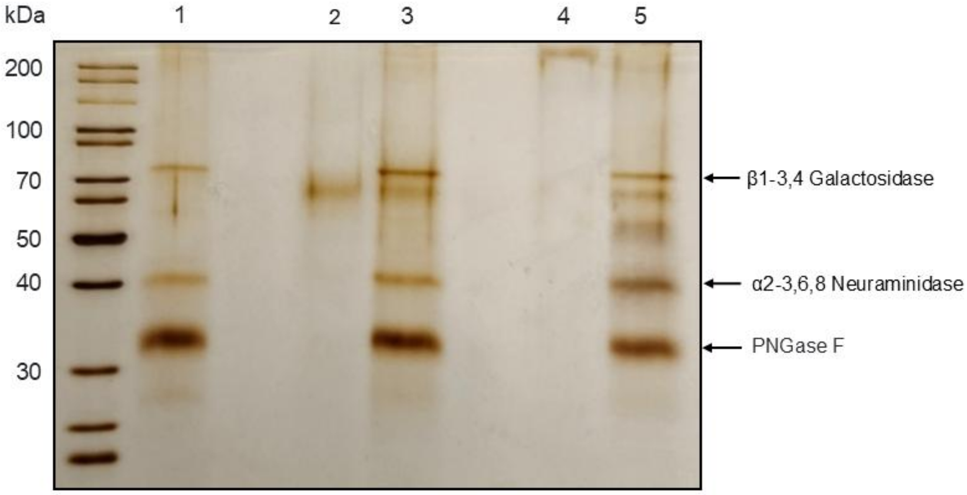

Fig. S1. (A) Full-size image of electrophoresis corresponding to (B) Fig. 2 from the manuscript. Lane descriptions: lane 1 – Protein Deglycosylation Mix alone (control); lane 2 – purified Yps3; lane 3 – Yps3 treated with Protein Deglycosylation Mix; lane 4 – purified Yps9; lane 5 – Yps9 treated with Protein Deglycosylation Mix. Proteins were separated by SDS-PAGE under reducing conditions using the Laemmli method with a 12% separating gel, and visualized by silver staining.

Tab. S1. Mass spectrometric identification of purified Yps. Two protein bands were excised from the SDS-PAGE gel: one from lane 2, corresponding to a position of approximately 60 kDa, and another from lane 4, located above 200 kDa (see Fig. S1). The excised bands were subjected to in-gel trypsin digestion, and the resulting peptides were analyzed using a Dionex Ultimate 3000 ultrahigh-performance liquid chromatography system coupled to an HCT Ultra ETDII mass spectrometer equipped with an electrospray ionization source (SC – sequence coverage).

Accession	Protein	Molecular mass [Da]	pI	Scores	Matches	Sequences	SC [%]
~60 kDa (line 2 in Fig. S1)							
QNG13114.1	YPS3 [Nakaseomyces glabratus]	58870	6.03	227	12	9	22
~200 kDa (line 4 in Fig. S1)							
QNG13119.1	YPS9 [Nakaseomyces glabratus]	56857	5.29	188	6	5	12

```
Name:  Yapsin9  Length:  521
MVKPLVLLSLLAYAQAYVKLDFTKTPGSDLAKRDVVDPEAAQLTFDKDQYIVEVAVGTPPQKVLLQIDTGSSDLFVIEES      80
NPYCKNNKNAPKKKDLESPGDDYPRTPNQVPKSDRTLDCKKYGFYNRNESSTFNSNGTDLFITYGDNGFVRGTWGTDSV     160
SVGNLNLSNLSIGVSPMTNTSTGILGVGLPGEESTFNYSSNVTGPSNYQYSNFPIRLKEEGLIEKIAYSIYLNETGSKYG     240
SILFGAVDHSKYQGPLYTFPLVNSKYKEGSDPFQFEITMNGVGLVGKTDNITLYDQKLPTLLDSGSTISLLPRDVADLVA     320
QQVNGTVDGKGNCIKLPKCPSKKDNQKLIFNFSGAEFSVNVTDFMEKHKGKCYLQFSALDGINFALLGDNFMNNVYTVFN     400
LDDKELSLAQANYNSSLKPDIEEIKDTVPSAVLAPQYYNTFSADPTATAVTGNIFAPEATMSMAAPANASRNSSLNSTFN     480
SSSNYSRVQMKKRTYPNSSSSLQANSAILIMIAAAITAMFL                                            560
................................................................................      80
................................................N.......N.......................     160
.....N..N...........................N...N...............................N.......     240
.................................................N..............................     320
...N..........................N........N........................................     400
.............N..............................................................N...N     480
...N.....................................                                            560

(Threshold=0.5)
----------------------------------------------------------------------
SeqName      Position  Potential   Jury    N-Glyc
                                 agreement result
----------------------------------------------------------------------
Yapsin9      129 NESS  0.6887     (8/9)    +
Yapsin9      137 NGTD  0.7863     (9/9)    +++
Yapsin9      166 NLSN  0.6887     (9/9)    ++
Yapsin9      169 NLSI  0.5798     (7/9)    +
Yapsin9      179 NTST  0.4116     (7/9)    -
Yapsin9      197 NYSS  0.7180     (9/9)    ++
Yapsin9      201 NVTG  0.6072     (7/9)    +
Yapsin9      233 NETG  0.6303     (8/9)    +
Yapsin9      290 NITL  0.7200     (9/9)    ++
Yapsin9      324 NGTV  0.6839     (9/9)    ++
Yapsin9      351 NFSG  0.5464     (6/9)    +
Yapsin9      360 NVTD  0.7427     (9/9)    ++
Yapsin9      414 NSSL  0.5071     (5/9)    +
Yapsin9      468 NASR  0.4451     (7/9)    -
Yapsin9      472 NSSL  0.3770     (6/9)    -
Yapsin9      476 NSTF  0.5119     (4/9)    +
Yapsin9      480 NSSS  0.5341     (4/9)    +
Yapsin9      484 NYSR  0.5670     (7/9)    +
Yapsin9      497 NSSS  0.4659     (5/9)    -
----------------------------------------------------------------------
```

Fig. S2. Prediction of potential N-glycosylation sites in Yps9 using the NetNGlyc server (Gupta et al., 2002). Identified sites are marked in red, indicating possible glycosylation motifs within the protein sequence.

A

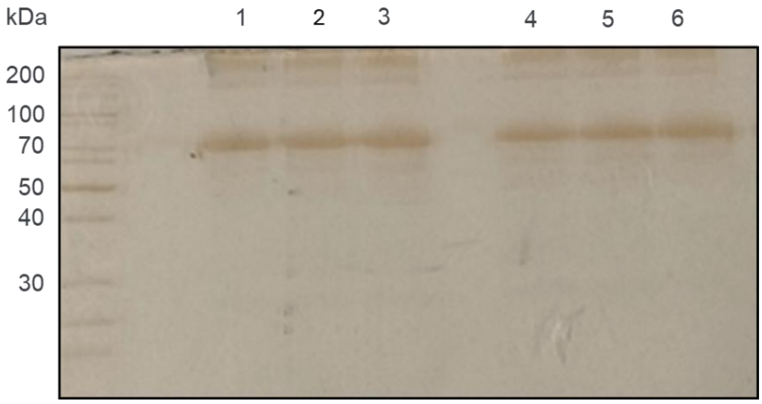

B

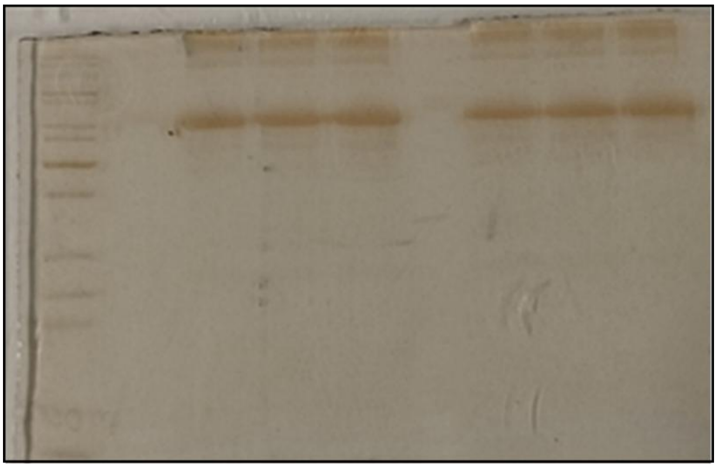

Fig S3. (A) Analysis of albumin (BSA) degradation by Yps3 and Yps9. BSA was incubated with Yps3 and Yps9 at a substrate to enzyme ratio of 50:1 overnight at pH 5.5 (lanes 2-3) or pH 7.0 (lanes 5-6). SDS-PAGE and silver staining were then performed to visualize the degradation products. A BSA sample incubated under the same conditions was used as a control. Molecular weight marker mixture is shown in the left lane. Lane 1: BSA, pH 5.5; lane 2: BSA with Yps3, pH 5.5; lane 3: BSA with Yps9, pH 5.5; lane 4: BSA, pH 7.0; lane 5: BSA with Yps3, pH 7.0; lane 6: BSA with Yps9, pH 7.0. (B) Full-size image of electrophoretic gel.

Tab. S2. Sequence identity matrix of 1-11 Yps from *C. glabrata* (11 sequences) and 1-10 Sap *C. albicans* (10 sequences), generated in UniProt using Clustal Omega. The matrix presents pairwise sequence identity percentages, providing an overview of the similarity between Yps and Sap protease families. The accession numbers of *C. glabrata* Yps sequences are A0A3G1PW07, Q6FVJ4, Q6FVI0, Q6FVH9, Q6FVH8, Q6FVH7, Q6FY32, Q6FVH6, Q6FVH5, Q6FVH4, Q6FVH3, while the accession numbers of *C. albicans* Sap sequences are P0CY26, P0CS83, P0CY28, Q5A8N2, P43094, P43095, P43096, O42778, O42779, Q5A651.

	Yps7	Sap9	Yps1	Yps11	Yps2	**Yps3**	Yps10	Yps4	Yps5	Yps9	Yps6	Yps8	Sap7	Sap10	Sap8	Sap5	Sap4	Sap6	Sap1	Sap2	Sap3
Yps7	100.0	24.6	25.3	19.8	22.2	**21.7**	21.0	22.5	23.2	21.5	22.1	21.3	24.1	20.7	23.5	22.6	22.6	22.6	23.0	22.6	21.2
Sap9	24.6	100.0	36.8	30.0	30.0	**28.6**	28.0	30.4	30.4	28.0	28.5	31.2	32.5	36.2	33.7	34.2	37.3	37.0	33.5	34.5	34.3
Yps1	25.3	36.8	100.0	37.4	39.7	**36.1**	38.1	38.3	38.1	36.6	39.2	37.8	35.3	30.5	32.7	31.9	30.7	31.1	33.2	32.3	33.2
Yps11	19.8	30.0	37.4	100.0	38.8	**38.2**	38.3	38.8	40.8	40.8	43.6	40.4	33.8	32.0	32.8	29.6	30.4	30.7	32.7	32.2	32.7
Yps2	22.2	30.0	39.7	38.8	100.0	**38.8**	42.5	40.8	39.2	41.8	43.3	39.6	34.6	30.3	31.4	30.6	32.6	32.0	30.9	33.3	32.3
Yps3	21.7	28.6	36.1	38.2	38.8	**100.0**	47.2	47.5	46.1	43.4	47.8	46.6	33.2	29.9	28.5	29.6	29.4	30.1	29.7	28.3	29.0
Yps10	21.0	28.0	38.1	38.3	42.5	**47.2**	100.0	48.8	49.1	48.0	50.6	51.0	33.1	29.8	30.9	29.4	29.1	29.1	30.3	30.7	30.0
Yps4	22.5	30.4	38.3	38.8	40.8	**47.5**	48.8	100.0	56.3	53.3	51.4	54.4	32.9	32.7	30.8	30.4	30.8	31.0	29.7	29.7	31.5
Yps5	23.2	30.4	38.1	40.8	39.2	**46.1**	49.1	56.3	100.0	50.7	52.9	53.3	37.0	32.0	34.5	31.5	31.9	32.4	31.1	31.9	31.8
**Yps9**	**21.5**	**28.0**	**36.6**	**40.8**	**41.8**	43.4	**48.0**	**53.3**	**50.7**	**100.0**	**56.6**	**53.8**	**32.4**	**33.1**	**31.3**	**31.4**	**31.1**	**31.4**	**32.3**	**33.5**	**32.0**
Yps6	22.1	28.5	39.2	43.6	43.3	**47.8**	50.6	51.4	52.9	56.6	100.0	55.5	36.4	30.9	32.9	32.2	32.8	32.5	32.0	32.3	33.1
Yps8	21.3	31.2	37.8	40.4	39.6	**46.6**	51.0	54.4	53.3	53.8	55.5	100.0	35.3	31.9	32.5	30.4	31.3	31.3	33.1	33.3	32.1
Sap7	24.1	32.5	35.3	33.8	34.6	**33.2**	33.1	32.9	37.0	32.4	36.4	35.3	100.0	30.1	34.4	31.8	31.7	33.1	31.7	32.9	32.9
Sap10	20.7	36.2	30.5	32.0	30.3	**29.9**	29.8	32.7	32.0	33.1	30.9	31.9	30.1	100.0	35.3	34.2	35.4	33.9	35.5	33.6	33.7
Sap8	23.5	33.7	32.7	32.8	31.4	**28.5**	30.9	30.8	34.5	31.3	32.9	32.5	34.4	35.3	100.0	47.3	48.2	47.9	54.2	54.7	55.8
Sap5	22.6	34.2	31.9	29.6	30.6	**29.6**	29.4	30.4	31.5	31.4	32.2	30.4	31.8	34.2	47.3	100.0	78.2	80.4	57.3	55.2	52.3
Sap4	22.6	37.3	30.7	30.4	32.6	**29.4**	29.1	30.8	31.9	31.1	32.8	31.3	31.7	35.4	48.2	78.2	100.0	90.9	59.3	58.1	56.5
Sap6	22.6	37.0	31.1	30.7	32.0	**30.1**	29.1	31.0	32.4	31.4	32.5	31.3	33.1	33.9	47.9	80.4	90.9	100.0	58.8	58.0	55.6
Sap1	23.0	33.5	33.2	32.7	30.9	**29.7**	30.3	29.7	31.1	32.3	32.0	33.1	31.7	35.5	54.2	57.3	59.3	58.8	100.0	73.4	71.0
Sap2	22.6	34.5	32.3	32.2	33.3	**28.3**	30.7	29.7	31.9	33.5	32.3	33.3	32.9	33.6	54.7	55.2	58.1	58.0	73.4	100.0	72.2
Sap3	21.2	34.3	33.2	32.7	32.3	**29.0**	30.0	31.5	31.8	32.0	33.1	32.1	32.9	33.7	55.8	52.3	56.5	55.6	71.0	72.2	100.0

```
CLUSTAL O(1.2.4) multiple sequence alignment


sp|P43096|CARP7_CANAX       MQRVLELLLLSSTALAVIGDGFIALPVHKLQAGEGSAHFPNRLPIFDVVNGVAKSVEDDV    60
tr|Q6FVI0|Q6FVI0_CANGA      ------------------------------------------------------------    0
tr|Q6FVH5|Q6FVH5_CANGA      ------------------------------------------------------------    0


sp|P43096|CARP7_CANAX       NQIIQPIFGNGIFSGGSIQGTHSGNGHSVKYEVSLPSSSAQKGSNGPSSTDNKDTDPSKT    120
tr|Q6FVI0|Q6FVI0_CANGA      ------------------------------------------------------------    0
tr|Q6FVH5|Q6FVH5_CANGA      ------------------------------------------------------------    0


sp|P43096|CARP7_CANAX       GFSLDDLMNSISTDFWNLIGLNKPPTSSDNGSKDADFTPSAVSQVEQPTSKSVESTAPGS    180
tr|Q6FVI0|Q6FVI0_CANGA      ------------MKFLLFAAVA-------QAYLQLDFERQ-----------------TAQ    24
tr|Q6FVH5|Q6FVH5_CANGA      ------MVKP--LVLLSLLAYA-------QAYVKLDFTKT-----------------PGS    28
                                          :  : .         :.  . **                     ..

sp|P43096|CARP7_CANAX       ASSASSSSSSEAASSSQPSEDSQPSSSANKKTGAFFLSLDNTQTLYTATLKVGSPAQEVQ    240
tr|Q6FVI0|Q6FVI0_CANGA      DVA----------LAKR--HTSNGV-----ANAMDVPIEQIGDLMYTVQLHVGTPPQNVT    67
tr|Q6FVH5|Q6FVH5_CANGA      DLA----------KRD----VVDPE-----A-----AQLTFDKDQYIVEVAVGTPPQKVL    64
                              :            .      :                   .  * . : **:* *:*

sp|P43096|CARP7_CANAX       VMIDTGSSDLWFISSGNSQCKVNG---------------------------GSIDCDKYG    273
tr|Q6FVI0|Q6FVI0_CANGA      VQLDTGSSDLWFPVASNPYCKRNAKLAPKKVK-ALPATGFATENDQVAKKLRTFDCDAFG    126
tr|Q6FVH5|Q6FVH5_CANGA      LQIDTGSSDLFVIEESNPYCKNNKNKAPKKKDLESPGDDYPRTPNQVPKSDRTLDCKKYG    124
                            : :*******:.   .*  ** *                             ::**. :*

sp|P43096|CARP7_CANAX       VFDKSKSSSWHDNK-TDYSISYYDGDKASGTMGQDNITFADGFSIENANFAVIDNTTSSI    332
tr|Q6FVI0|Q6FVI0_CANGA      LFNSSMSSSFKSNDSSEFFVKYEDGTYASGMWGTDTFKLN-HHNVSNITFALANIANASM    185
tr|Q6FVH5|Q6FVH5_CANGA      FYNRNESSTFNSNG-TDLFITYGDNGFVRGTWGTDSVSVG-NLNLSNLSIGVSPMTNTST    182
                            .:: . **:::.*  ::  :.* *.  . *  * *....    .:.* .:.:   :.:*

sp|P43096|CARP7_CANAX       GVFGVGYPELEAV------------KSKYTNLPFAMKEQNLIAKVAYSLYLDSRDAVQGY    380
tr|Q6FVI0|Q6FVI0_CANGA      GVLGVGFPAEETTDDPSPGSLIDKEHYQYDNFPIALKRTRTIKKVSYSIFLNDTNSKKGV    245
tr|Q6FVH5|Q6FVH5_CANGA      GILGVGLPGEESTFNYS-SNVTGPSNYQYSNFPIRLKEEGLIEKIAYSIYLNETGSKYGS    241
                            *::*** *  *:.            : :* *:*: :*.   * *::**::*:. .:  *

sp|P43096|CARP7_CANAX       ILFGGIDHAFYTGDLKAFDIVQCNDKYVYSQIPLTSVASSLNNYTNAYGLPAGSNHPKVG    440
tr|Q6FVI0|Q6FVI0 CANGA      ILFGGVDHSKYQGTLWTVPMVNNLLT--LNQTTTSRPEI----TLNGLGFRDDK------    293
```

Fig. S4. Comparison of amino acid sequence of *C. albicans* Sap7 (accession number P43096), *C. glabrata* Yps3 (accession number Q6FVI0), and *C. glabrata* Yps9 (accession number Q6FVH5), and aligned using Clustal Omega.

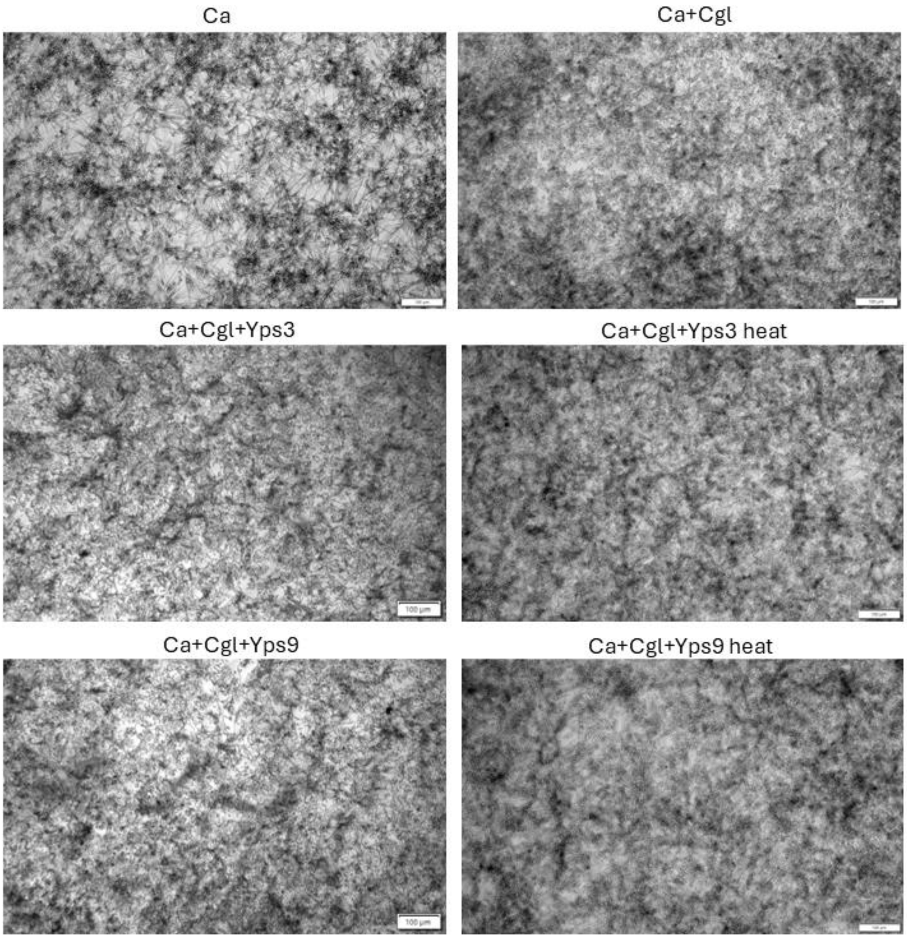

Fig. S5. Representative transmitted light microscopy images of biofilms. Images show the morphology of mixed (*C. albicans–C. glabrata*) biofilms treated with native or heat-denatured Yps3 and Yps9, as well as the untreated *C. albicans* monospecies control. Biofilms were cultured for 24 hours in the presence or absence of purified Yps proteins and imaged after PBS washing.

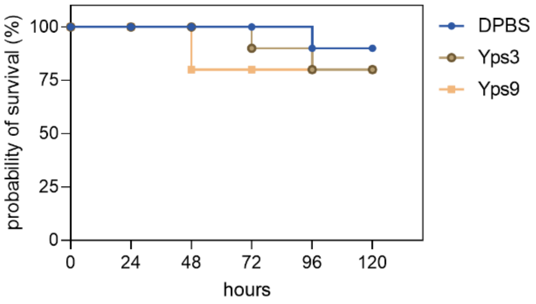

Fig. S6. Assessment of the cytotoxicity of Yps proteins in *Galleria mellonella* larvae. Larvae were injected with 1 µg of Yps3 or Yps9 in 10 µl DPBS, and their survival was monitored for 96 hours to evaluate potential toxic effects of the proteases. Larvae injected with DPBS served as a control. Representative data are shown in the graph, with the experiment performed in two independent biological replicates, each group containing ten larvae.
